# Development of a quality indicator set to measure and improve quality of ICU care for patients with traumatic brain injury

**DOI:** 10.1186/s13054-019-2377-x

**Published:** 2019-03-22

**Authors:** Jilske A. Huijben, Eveline J. A. Wiegers, Nicolette F. de Keizer, Andrew I. R. Maas, David Menon, Ari Ercole, Giuseppe Citerio, Fiona Lecky, Lindsay Wilson, Maryse C. Cnossen, Suzanne Polinder, Ewout W. Steyerberg, Mathieu van der Jagt, Hester F. Lingsma, the Delphi panel, Marcel Aries, Rafael Badenes, Albertus Beishuizen, Federico Bilotta, Arturo Chieregato, Emiliano Cingolani, Giuseppe Citerio, Maryse Cnossen, Mark Coburn, Jonathan P. Coles, Mark Delargy, Bart Depreitere, Ari Ercole, Hans Flaatten, Volodymyr Golyk, Erik Grauwmeijer, Iain Haitsma, Raimund Helbok, Cornelia Hoedemaekers, Bram Jacobs, Korné Jellema, Lars-Owe D. Koskinen, Andrew I. R. Maas, Marc Maegele, Maria Cruz Martin Delgado, David Menon, Kirsten Møller, Rui Moreno, David Nelson, Annemarie W. Oldenbeuving, Jean-Francois Payen, Suzanne Polinder, Jasmina Pejakovic, Gerard M. Ribbbers, Rolf Rossaint, Guus Geurt Schoonman, Luzius A. Steiner, Nino Stocchetti, Fabio Silvio Taccone, Riikka Takala, Olli Tenovuo, Eglis Valeinis, Walter M. van den Bergh, Mathieu van der Jagt, Thomas van Essen, Nikki van Leeuwen, Michael H. J. Verhofstad, Pieter E. Vos, Lindsay Wilson

**Affiliations:** 1 Department of Public Health, Center for Medical Decision Making, Erasmus MC University Medical Center Rotterdam, Rotterdam, The Netherlands; 2 Department of Medical Informatics, Amsterdam Public Health research institute, Academic Medical Center, University of Amsterdam, Amsterdam, The Netherlands; 3 Department of Neurosurgery, Antwerp University Hospital and University of Antwerp, Edegem, Belgium; 4 Division of Anaesthesia, University of Cambridge, Addenbrooke’s Hospital, Cambridge, UK; 5 School of Medicine and Surgery, University of Milan-Bicocca, Milan, Italy; 6 Neuro-Intensive Care, Department of Emergency and Intensive Care, San Gerardo Hospital, ASST, Monza, Italy; 7 Centre for Urgent and Emergency Care Research, School of Health and Related Research, University of Sheffield, Sheffield, UK; 8 Division of Psychology, University of Stirling, Stirling, UK; 9 Department of Biomedical Data Sciences, Leiden University Medical Center, Leiden, The Netherlands; 10 Department of Intensive Care Adults, Erasmus MC University Medical Center, Rotterdam, The Netherlands

**Keywords:** Quality indicators, Benchmarking, Traumatic brain injury, Intensive care unit, Trauma registry, Quality of care

## Abstract

**Background:**

We aimed to develop a set of quality indicators for patients with traumatic brain injury (TBI) in intensive care units (ICUs) across Europe and to explore barriers and facilitators for implementation of these quality indicators.

**Methods:**

A preliminary list of 66 quality indicators was developed, based on current guidelines, existing practice variation, and clinical expertise in TBI management at the ICU. Eight TBI experts of the Advisory Committee preselected the quality indicators during a first Delphi round. A larger Europe-wide expert panel was recruited for the next two Delphi rounds. Quality indicator definitions were evaluated on four criteria: validity (better performance on the indicator reflects better processes of care and leads to better patient outcome), feasibility (data are available or easy to obtain), discriminability (variability in clinical practice), and actionability (professionals can act based on the indicator). Experts scored indicators on a 5-point Likert scale delivered by an electronic survey tool.

**Results:**

The expert panel consisted of 50 experts from 18 countries across Europe, mostly intensivists (*N* = 24, 48%) and neurosurgeons (*N* = 7, 14%). Experts agreed on a final set of 42 indicators to assess quality of ICU care: 17 structure indicators, 16 process indicators, and 9 outcome indicators. Experts are motivated to implement this finally proposed set (*N* = 49, 98%) and indicated routine measurement in registries (*N* = 41, 82%), benchmarking (*N* = 42, 84%), and quality improvement programs (*N* = 41, 82%) as future steps. Administrative burden was indicated as the most important barrier for implementation of the indicator set (*N* = 48, 98%).

**Conclusions:**

This Delphi consensus study gives insight in which quality indicators have the potential to improve quality of TBI care at European ICUs. The proposed quality indicator set is recommended to be used across Europe for registry purposes to gain insight in current ICU practices and outcomes of patients with TBI. This indicator set may become an important tool to support benchmarking and quality improvement programs for patients with TBI in the future.

**Electronic supplementary material:**

The online version of this article (10.1186/s13054-019-2377-x) contains supplementary material, which is available to authorized users.

## Background

Traumatic brain injury (TBI) causes an enormous health and economic burden around the world [1]. Patients with moderate and severe TBI are at high risk for poor outcomes and often require intensive care unit (ICU) admission. In these patients, evidence-based treatment options are scarce and large differences in outcome and daily ICU practice exist [2–5].

Research to establish more evidence-based and thereby uniform treatment policies for patients with TBI has high priority. Still, breakthrough intervention strategies are scarce [6] and guideline recommendations remain limited. Therefore, new strategies, such as precision medicine and routine quality measurement, are being explored to drive research and clinical practice forward [1]. Routine quality measurement using appropriate indicators can guide quality improvement, for example, through identifying best practices and internal quality improvement initiatives. The potential of quality indicators to improve care has already been demonstrated in other clinical areas [7], in other ICU populations like sepsis [8] or stroke patients [9], and in children with TBI [10, 11].

However, there are also examples of quality indicators that do not positively affect the quality of care. This may be for various reasons, such as lack of validity and reliability, poor data quality, or lack of support by clinicians [12–14]. Deploying poor indicators has opportunity costs due to administrative burden while distorting healthcare priorities. An evaluation of a putative quality indicator is inherently multidimensional, and when used to identify best practice or benchmark hospitals, validity and reliability and uniform definitions are all equally important [15, 16].

Although some quality indicator sets for the general ICU exist [17, 18], there are no consensus-based quality indicators specific for the treatment of adult patients with TBI. Delphi studies have been proposed as a first step in the development of quality indicators [19]. The systematic Delphi approach gathers information from experts in different locations and fields of expertise to reach group consensus without groupthink [19], an approach which aims to ensure a breadth of unbiased participation.

The aim of this study was to develop a consensus-based European quality indicator set for patients with TBI at the ICU and to explore barriers and facilitators for implementation of these quality indicators.

## Methods

This study was part of the Collaborative European NeuroTrauma Effectiveness Research in Traumatic Brain Injury (CENTER-TBI) project [20].

An Advisory Committee (AC) was convened, consisting of 1 neurosurgeon (AM), 3 intensivists (MJ, DM, GC), 1 emergency department physician (FL), and 3 TBI researchers (HL, ES, LW) from 5 European countries. The AC’s primary goals were to provide advice on the recruitment of the Delphi panel, to monitor the Delphi process, and to interpret the final Delphi results. During a face-to-face meeting (September 2017), the AC agreed that the Delphi study would initially be restricted to Europe, recruit senior professionals as members of the Delphi panel, and focus on the ICU. The restriction to a European rather than a global set was motivated by substantial continental differences in health funding systems, health care costs, and health care facilities. The set was targeted to be generalizable for the whole of Europe and therefore included European Delphi panelists. The AC agreed to target senior professionals as Delphi panelists as they were expected to have more specialized and extensive clinical experience with TBI patients at the ICU. The AC decided to focus the indicator set on ICU practice, since ICU mortality rates are high (around 40% in patients with severe TBI [21]), large variation in daily practice exists [2–5, 22], and detailed data collection is generally more feasible in the ICU setting due to available patient data management systems or electronic health records (EHRs). We focused on adult patients with TBI.

### Delphi panel

The AC identified 3 stakeholder groups involved in ICU quality improvement: (1) clinicians (physicians and nurses) primary responsible for ICU care, (2) physicians from other specialties than intensive care medicine who are regularly involved in the care of patients with TBI at the ICU, and (3) researchers/methodologists in TBI research. It was decided to exclude managers, auditors, and patients as stakeholders, since the completion of the questionnaires required specific clinical knowledge. Prerequisites to participate were a minimum professional experience of 3 years at the ICU or in TBI research. Stakeholders were recruited from the personal network of the AC (also through social media), among the principal investigators of the CENTER-TBI study (contacts from more than 60 NeuroTrauma centers across 22 countries in Europe) [20], and from a European publication on quality indicators at the ICU [18]. These experts were asked to provide additional contacts with sufficient professional experience.

### Preliminary indicator set

Before the start of the Delphi process, a preliminary set of quality indicators was developed by the authors and the members of the AC, based on international guidelines (Brain Trauma Foundation [23] and Trauma Quality Improvement Program guidelines [24]), ICU practice variation [3–5], and clinical expertise (Additional file 1: Questionnaire round 1). Quality indicators were categorized into structure, process, and outcome indicators [25]. Overall, due to the absence of high-quality evidence on which thresholds to use in TBI management, we refrained from formulating quality indicators in terms of thresholds. For example, we did not use specific carbon dioxide (CO_2_) or intracranial pressure (ICP) thresholds to define quality indicators for ICP-lowering treatments.

### Indicator selection

The Delphi was conducted using online questionnaires (Additional files 1, 2, and 3). In the first round, the AC rated the preliminary quality indicators on four criteria: validity, discriminability (to distinguish differences in center performance), feasibility (regarding data collection required), and actionability (to provide clear directions on how to change TBI care or otherwise improve scores on the indicator) [26–30] (Table 1). We used a 5-point Likert scale varying from strongly disagree (1) to strongly agree (5). Additionally, an “I don’t know” option was provided to capture uncertainty. Agreement was defined as a median score of 4 (agreement) or 5 (strong agreement) on all criteria. Disagreement was defined as a median score below 4 on at least one of the four criteria [31, 32]. Consensus was defined as an interquartile range (IQR) ≤ 1 (strong consensus) on validity—since validity is considered the key characteristic for a useful indicator [19]—and IQR ≤ 2 (consensus) on the other criteria [31, 32]. Criteria for rating the indicators and definitions of consensus remained the same during all rounds. The AC was able to give recommendations for indicator definitions at the end of the questionnaire. Indicators were excluded for the second Delphi round when there was consensus on disagreement on at least one criterion, unless important comments for improvement of the indicator definition were made. Such indicators with improved definitions were rerated in the next Delphi round.

**Table 1:** Selection criteria used to rate the quality indicators

Criteria	Definition
Validity	It is likely that better performance on the indicator reflects better processes of care and leads to a better patient outcome
Feasibility	Measurement of the indicator is feasible (data for the indicator are available or easy to obtain)
Discriminability	It is expected that there is variability in clinical practice
Actionability	The indicator can be used to improve quality of care, and professionals can act on it

These criteria were used to rate each quality indicator during all Delphi rounds [26–30]

In the second round, the remaining indicators were sent to a larger group of experts. The questionnaire started with a description of the goals of the study, and some characteristics of experts were asked. Experts had the possibility to adapt definitions of indicators at the end of a group of indicators on a certain topic (domain). Indicators were included in the final set when there was agreement and consensus, excluded when there was disagreement and consensus, and included the next round when no consensus was reached or important comments to improve the indicator definitions were given. As many outcome scales exist for TBI, like the Glasgow Outcome Scale - Extended (GOSE), Coma Recovery Scale - Revised (CSR-R), and Rivermead Post-Concussion Symptoms Questionnaire (RPQ), a separate ranking question was used to determine which outcome scales were preferred (or most important) to use as outcome indicators—to avoid an extensive outcome indicator set (Additional file 2, question outcome scales). Outcome scales that received the highest ratings (top 3) were selected for round 3 and rated as described above. Finally, exploratory questions were asked for which goals or reasons experts would implement the quality indicators. We only selected experts for the final round that completed the full questionnaire.

In the last round, the expert panel was permitted only to rate the indicators, but could not add new indicators or suggest further changes to definitions. Experts received both qualitative and quantitative information on the rating of indicators (medians and IQRs) from round 2 for each individual indicator. Indicators were included in the final set if there was both agreement and consensus. Final exploratory questions were asked regarding the barriers and facilitators for implementation of the indicator set. For each Delphi round, three automated reminder emails and two personal reminders were sent to the Delphi participant to ensure a high response rate.

### Statistical analysis

Descriptive statistics (median and interquartile range) were calculated to determine which indicators were selected for the next round and to present quantitative feedback (median and min-max rates) in the third Delphi round. “I don’t know” was coded as missing. A sensitivity analysis after round 3 was performed to determine the influence of experts from Western Europe compared with other European regions on indicator selection (in- or exclusion in the final set). Statistical analyses were performed using the R statistical language [33]. Questionnaires were developed using open-source LimeSurvey software [34]. In LimeSurvey, multiple online questionnaires can be developed (and send by email), the response rates can be tracked, and questionnaire scores or responses can easily be exported to a statistical program.

## Results

### Delphi panel

**Fig. 1:**
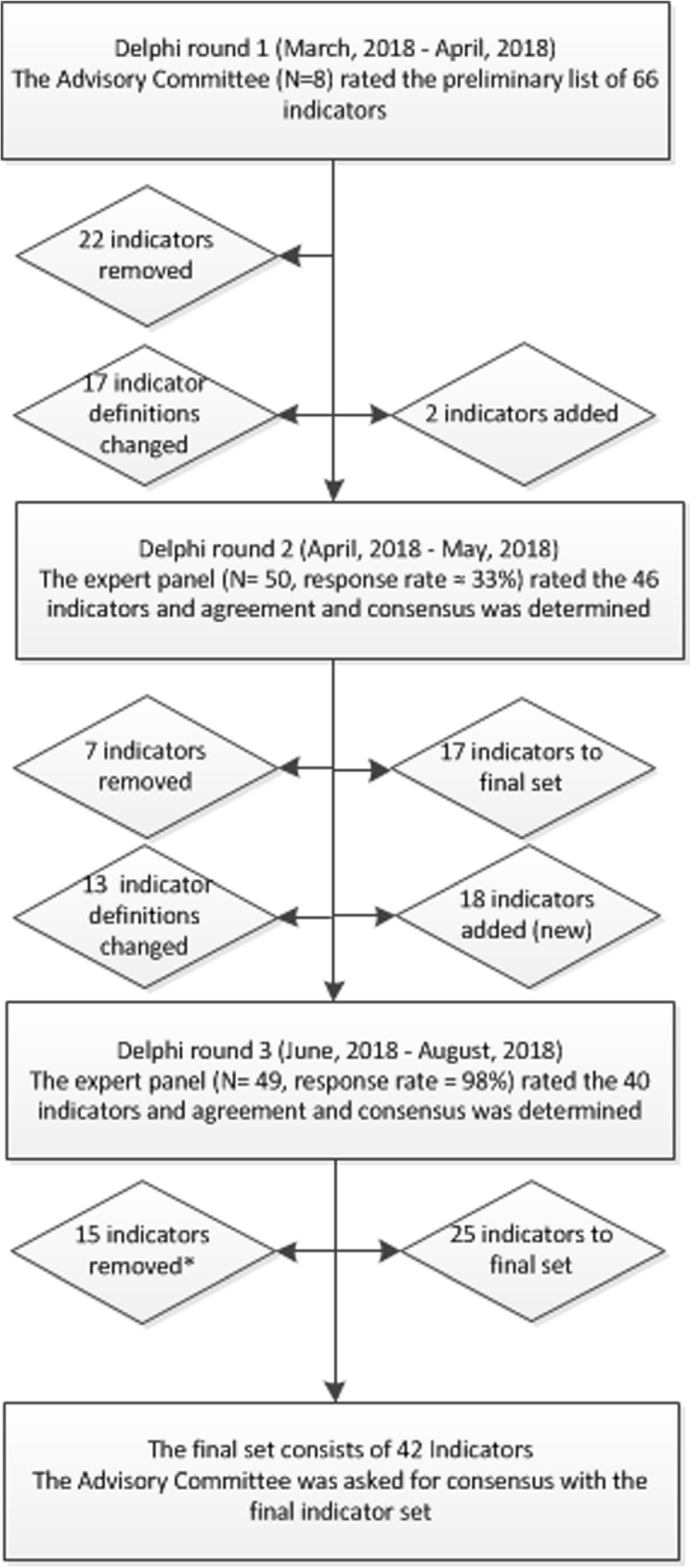
Overview of the Delphi process. Overview of the Delphi process: time frame, experts’ involvement, and indicator selection; *8 indicators were removed based on the sensitivity analyses. The left site of the figure shows the number of indicators that were removed after disagreement and consensus with no comments to improve definitions. In addition, the number of changed indicator definitions is shown. The right site of the figure shows the number of newly proposed indicators (that were rerated in the next Delphi round) and the number of indicators that were included in the final indicator set. After round 2, 17 indicators were included in the final set (and removed from the Delphi process), and after round 3, 25 indicators were included in the final set—a total of 42 indicators. The agreement was defined as a median score of 4 (agreement) or 5 (strong agreement) on all four criteria (validity, feasibility, discriminability, and actionability) to select indicators. The disagreement was defined as a median score below 4 on at least one of the four criteria. The consensus was defined as an interquartile range (IQR) ≤ 1 (strong consensus) on validity—since validity is considered the key characteristic for a useful indicator [19]—and IQR ≤ 2 (consensus) on the other criteria

The Delphi rounds were conducted between March 2018 and August 2018 (Fig. 1). Approximately 150 experts were invited for round 2, and 50 experts from 18 countries across Europe responded (≈33%). Most were intensivists (*N* = 24, 48%), followed by neurosurgeons (*N* = 7, 14%), neurologists (*N* = 5, 10%), and anesthesiologist (*N* = 5, 10%) (Table 2). Most of the experts indicated to have 15 years or more experience with patients with TBI at the ICU or another department (*N* = 25, 57%). Around half of the experts indicated that they had primary responsibility for the daily practical care of patients with TBI at the ICU (*N* = 21, 47%). Experts were employed in 37 centers across 18 European countries: mostly in Western Europe (*N* = 26, 55%). Most experts were from academic (*N* = 37, 84%) trauma centers in an urban location (*N* = 44, 98%). Almost all experts indicated the availability of EHRs in their ICU (*N* = 43, 96%). Thirty-one experts (63%) participated in the CENTER-TBI study. The response rate in round 3 was 98% (*N* = 49).

**Table 2:** Baseline characteristics Delphi panel

	Number	Percent
Total number of Delphi panelists	50	100
Total number of participating centers	37	100
Gender (*N* = 50)		
Male	40	80
Female	10	20
Profession (*N* = 50)		
Neurosurgeon	7	14
Intensivist	24	48
Neurologist	5	10
Anesthesiologist	5	10
Trauma surgeon	2	4
Rehabilitation specialist	3	6
Methodologist/researcher in TBI	3	6
Neurophysiologist	1	2
Number of years of professional experience at the ICU ^a^(*N* = 44)		
3–5 years	4	9
5–10 years	8	18
10–15 years	7	16
> 15 years	25	57
Primary responsible/in charge for the daily care of patients with TBI at the ICU ^a^(*N* = 45)		
Yes	21	47
No	24	53
Location ^b^(*N* = 50)		
Northern Europe	6	12
Western Europe	28	56
UK	5	10
Southern Europe	8	16
Eastern Europe	2	4
Baltic States	1	2
Center (*N* = 44)		
Academic	37	84
Nonacademic	7	16
Center location ^c^(*N* = 45)		
Urban	44	98
Suburban	1	2
Trauma designation ^d^(*N* = 45)		
Level I	31	69
Level II	1	2
Level III	7	15
Our center is not officially designated as a trauma center	3	7
Our country does not explicitly designate trauma centers	3	7
Electronic patient records ^a^(*N* = 45)		
Yes	43	96
No	2	4
Participation in CENTER-TBI study (*N* = 49)		
Yes	31	63
No	18	42

Level II trauma center: A level II trauma center provides comprehensive trauma care in either a population-dense area in which a level II trauma center may supplement the clinical activity and expertise of a level I institution or occur in less population-dense areas. In the latter case, the level II trauma center serves as the lead trauma facility for a geographic area when a level I institution is not geographically close enough to do so. It is characterized by 24-h in-house availability of an attending surgeon and the prompt availability of other specialties (e.g., neurosurgeon, trauma surgeon). Level III trauma center: A level III trauma center has the capacity to initially manage the majority of injured patients and have transfer agreements with a level I or II trauma center for seriously injured patients whose needs exceed the facility’s resources

*TBI* traumatic brain injury, *CENTER-TBI study* Collaborative European NeuroTrauma Effectiveness Research in Traumatic Brain Injury study, *ICU* intensive care unit

^a^Only asked to those who answered clinician as a profession

^b^Location is based on United Nations geoscheme: Northern Europe = Norway (1), Sweden (2), Finland (2), and Denmark (1); Western Europe = Austria(1), Belgium (3), France (1), Germany (4), Switzerland (1), and The Netherlands (18); the UK and Ireland (5), Southern Europe = Portugal (1), Italy (5), and Spain (2); Eastern Europe = Ukraine (1), Serbia (1); Baltic States = Latvia (1)

^c^Urban: an hospital location very near to a city and situated in a crowded area

Suburban: between urban and rural (an hospital location in or very near to the countryside in an area that is not crowded.)

^d^Level I trauma center: A regional resource center that generally serves large cities or population-dense areas. A level I trauma center is expected to manage large numbers of severely injured patients (at least 1200 trauma patients annually or have 240 admissions with an Injury Severity Score of more than 14). It is characterized by 24-h in-house availability of an attending surgeon and the prompt availability of other specialties (e.g., neurosurgeon, trauma surgeon)

### Indicator selection

The first Delphi round started with 66 indicators (Fig. 1). In round 1, 22 indicators were excluded. The main reason for exclusion was poor agreement (median < 4) on all criteria except discriminability (Additional file 4). Round 2 started with 46 indicators; 17 were directly included in the final set and 7 were excluded, mainly due to a poor agreement (median < 4) on actionability and poor consensus (IQR > 1) on validity. Round 3 started with 40 indicators; 25 indicators were included in the final set. Exclusion of 8 indicators was based on the sensitivity analysis (no consensus in Western Europe versus other European regions) and 7 indicators had low agreement on actionability or no consensus on validity or actionability. During the full Delphi process, 20 new indicators were proposed, and 30 definitions were discussed and/or modified.

The final quality indicator set consisted of 42 indicators on 13 clinical domains (Table 3), including 17 structure indicators, 16 process indicators, and 9 outcome indicators. For the domains “precautions ICP monitoring,” “sedatives,” “osmotic therapies,” “seizures,” “fever,” “coagulopathy,” “respiration and ventilation,” and “red blood cell policy,” no indicators were included in the final set.

**Table 3:** Finally proposed set of clinical quality indicators in traumatic brain injury at the ICU

Domain	Indicators
Protocol	
	1. Structure: The existence of a protocol including specific guidelines (like the BTF guidelines or institutional guidelines) for traumatic brain injury patients (yes/no)
	2. Structure: The presence of (some form of) regular audits to check guideline adherence in general at the intensive care unit (ICU) (yes/no) Extra: Audits do not have to be specific for TBI
	3. Structure: The presence of a dedicated person(s) to oversee guidelines development and maintenance, including those for patients with TBI, at the ICU (yes/no)
Intensive care unit	
	4. Structure: The presence of a step-down unit where patients can still be monitored 24/7, but less intensively than at the ICU (yes/no) Extra: A facility in-between ICU and ward. It is often used for patients who improved at the intensive care and no longer need the intensity of ICU care, but are also not well enough to be cared for at the ward. The care provided in step down beds is less intensive than the care provided at the ICU but more intensive than ward care
	5. Structure: Does your hospital have a dedicated/specialized neurocritical care unit? (yes/no)
	6. Structure: The availability of operating rooms 24 h per day (yes/no)
	7. Process: Median accident-to-ICU-admission time (process) Extra: Time of the accident/injury to ICU-door-time
Staff	
	8. Structure: A daily meeting between intensivist and neurosurgeon to discuss patients with TBI at the ICU (yes/no)
	9. Structure: Availability of a neurosurgeon (staff) 24/7 within 30 min after the call (yes/no)
	10. Structure: Total number of disciplines (i.e., neurologist, physiotherapy, occupational therapy) involved during ICU stay
	11. Structure: Certified intensivist present in person 7 days a week during at least day-time (yes/no)
	12. Structure: Intensivist to ICU bed ratio
	13. Structure: ICU nurse to ICU bed ratio
	14. Process: Number of visits by a neurosurgeon/ total number of ICU days in patients with TBI
CT scanning	
	15. Structure: 24/7 availability of a CT scan and radiologist review (yes/no)
ICP monitoring	
	16. Structure: 24/7 availability of a certified person at your center that can insert an ICP monitor within 2 h after admission at the ICU (yes/no)
	17. Process: Number of severe (GCS 3–8) TBI patients with ICP monitoring/number of severe TBI patients at the ICU
	18. Outcome: Number of EVD infections in patients with TBI/total number of patients with TBI at the ICU with an EVD inserted Extra: Only for centers that use ventricular catheters
Deep venous thrombosis (DVT)	
	19. Process: Number of patients with TBI that receive any DVT prophylaxis/total number of patients with TBI at the ICU Extra: Timing (application of prophylaxis in days from the injury) and type of DVT prophylaxis (mechanical and/or pharmaceutical) can be registered as well
	20. Process: Number of patients that receive pharmaceutical prophylaxis with low molecular weight heparins/total number of TBI patients admitted to the ICU Extra: This QI is about the choice of prophylaxis (low molecular weight heparin), not about timing
	21. Process: Number of patients with TBI that receive mechanical DVT prophylaxis (e.g., stockings) initiated within 6 h/total number of patients with TBI at the ICU with the possibility to receive stockings Extra: Exclude patients with leg fractures
Glucose and nutrition	
	22. Structure: Do you have a protocol for glucose management available for patients with TBI at your ICU? yes/no
	23. Process: Number of TBI patients with basal full caloric replacement within 5 to 7 days post-injury/number of TBI patients at the ICU
	24. Process: Number of TBI patients with start of (early) enteral nutrition within 72 h/number of patients with enteral feeding during ICU stay
	25. Outcome: Number of TBI patients with any blood glucose above 10 mmol/L (180 mg/dL, hyperglycemia)/total number of patients with TBI at the ICU Extra: Other values are not necessarily good, only detection of extreme cases
	26. Outcome: Number of TBI patients with any blood glucose below 4 mmol/L (hypoglycemia)/total number of patients with TBI at the ICU Extra: Other values are not necessarily good, only detection of extreme cases
Surgery	
	27. Structure: The presence of a protocol/institutional guideline that provide indications for surgery with SDH an EDH (yes/no)
	28. Process: Median door-to-operation time for acute operation of SDH and EDH with surgical indication
Allied health professional	
	29. Process: Number of patients with a support plan (e.g., rehabilitation) after ICU discharge/number of patients discharged from the ICU Extra: plan consists of physio-, speech-, and occupational therapist goals during hospital stay
	30. Process: Number of patients with TBI visited daily by a physiotherapist during ICU stay/total number of patients with TBI at the ICU
Assessment scales at the ICU	
	31. Structure: Information on prognosis is discussed with family by one of the treating physicians (ICU physician or neurosurgical physician) at least once during ICU stay
	32. Process: Number of assessments of motor scores of the GCS/total number of ICU days in patients with TBI
	33. Process: Number of assessments of pupillary responses/total number of ICU days in patients with TBI
	34. Process: Number of assessments of delirium presence with validated screening tool in conscious TBI patients/total number of ICU days in conscious TBI patients
In-hospital outcomes	
	35. Outcome: Number of ICU-deaths among patients with TBI/total number of ICU-admitted patients with TBI
	36. Outcome: Incidence of ventilator-associated pneumonia (VAP) in patients with TBI/total number of TBI patients with mechanical ventilation at the ICU Extra: Pneumonia defined as “the presence of new lung infiltrate plus clinical evidence that the infiltrate is of an infectious origin, which includes the new onset of fever, purulent sputum, leukocytosis, and a decline in oxygenation,”. VAP is defined as pneumonia occurring > 48 h after endotracheal intubation [46]
	37. Outcome: Number of TBI patients with decubitus grade 2 or higher at the ICU/number of TBI patients at the ICU Extra (also register the grade): Grade 1: Pressure zone with redness that does not blanch with fingertip pressure, with the skin still intact Grade 2: Decubitus ulcer (pressure sore) with skin erosion, blister, partial loss of the epidermis and/or dermis, or skin loss Grade 3: Decubitus ulcer (pressure sore) with loss of all skin layers and damage or necrosis of the subcutaneous tissue, which may extend down to the underlying fascia Grade 4: Decubitus ulcer (pressure sore) with necrosis of the muscle, bone, or supportive structures such as tendons or joint capsules
	38. Outcome: Number of patients with TBI with severe sepsis or septic shock/total number of patients with TBI at the ICU Extra: Sepsis should be defined as life-threatening organ dysfunction caused by a dysregulated host response to infection. For clinical operationalization, organ dysfunction can be represented by an increase in the Sequential [sepsis-related] Organ Failure Assessment (SOFA) score of 2 points or more, which is associated with an in-hospital mortality greater than 10%. The septic shock should be defined as a subset of sepsis in which particularly profound circulatory, cellular, and metabolic abnormalities are associated with a greater risk of mortality than with sepsis alone. Patients with septic shock can be clinically identified by a vasopressor requirement to maintain a mean arterial pressure of 65 mmHg or greater and serum lactate level greater than 2 mmol/L (> 18 mg/dL) in the absence of hypovolemia [47]
After discharge or follow-up outcomes	
	39. Process: Number of patients with TBI receiving follow-up by a specialist within 2 months after discharge/total number of patients with TBI discharged (not in rehab clinic)
	40. Process: Number of patients with neuropsychological testing at hospital discharge/number of patients with TBI discharged from the hospital
Outcome scales at 6 months	
	41. Outcome: The median score of the GOSE from all patients with TBI at 6 months/number of patients with TBI discharged from the ICU and alive at 6 months
	42. Outcome: The median score of the SF-36 from all patients with TBI at 6 months/number of patients with TBI discharged from the ICU and alive at 6 months

The final set of indicators after the Delphi rounds per domain. All outcome indicators will be adjusted for case-mix

*EDH* epidural hematoma, *GCS* Glasgow Coma Scale, *GOSE* Glasgow Coma Scale – Extended, *ICU* intensive care unit *SDH* subdural hematoma, *SF-36* 36-item short form survey

Experts proposed changing the names of the “short-term outcomes” and “long-term outcomes” domains to “in-hospital outcomes” and “after discharge or follow-up outcomes.” In round 2, the Glasgow Outcome Coma Scale Extended (GOSE), quality of life after brain injury (Qolibri), and short form health survey (SF-36) were rated the best outcome scales. However, the Qolibri was excluded in round 3 as an outcome indicator, since there was no consensus in the panel on its validity to reflect the quality of ICU care. The majority of experts (*N* = 14, 28%) indicated that the outcome scales should be measured at 6 months, but this was closely followed by experts that indicated both at 6 and 12 months (*N* = 13, 26%).

### Barriers and facilitators for implementation

Almost all experts indicated that the indicator set should be used in the future (*N* = 49, 98%). One expert did not believe an indicator set should be used at all, because it would poorly reflect the quality of care (*N* = 1, 2%).

**Fig. 2:**
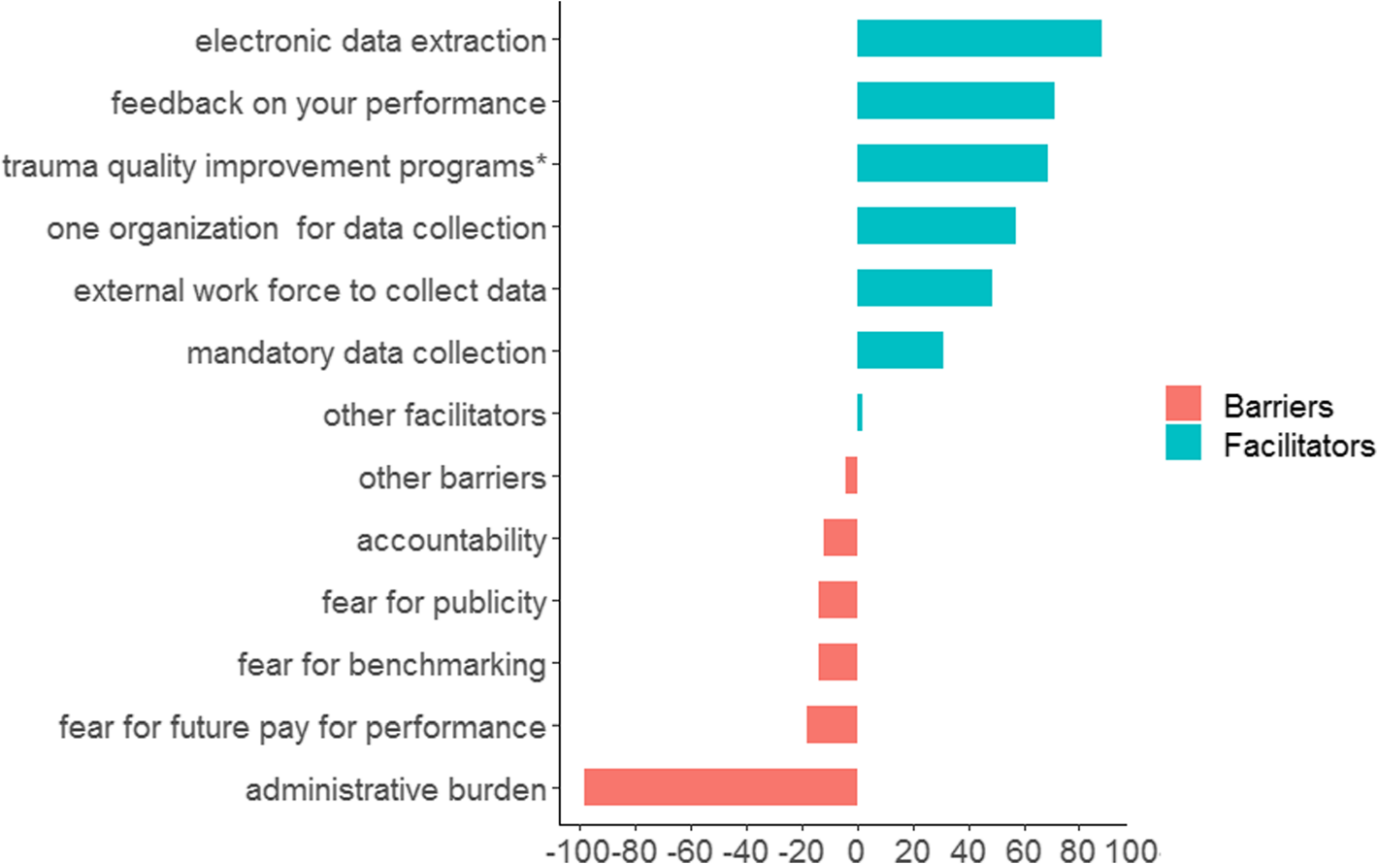
Facilitators or barriers for implementation of the quality indicator set. Percentage of experts that indicated a certain facilitator or barrier for implementation of the quality indicator set. Other indicated facilitator was “create meaningful uniform indicators.” Other indicated barriers were “gaming” (*N* = 1, 2%) and “processes outside of ICU (e.g., rehabilitation) are hard to query.” *Participation in trauma quality improvement program

The majority of experts indicated that the set could be used for registry purposes (*N* = 41, 82%), assessment of adherence to guidelines (*N* = 35, 70%), and quality improvement programs (*N* = 41, 82%). Likewise, the majority of experts indicated that the indicator set could be used for benchmarking purposes (*N* = 42, 84%); both within and between centers. Pay for performance was rarely chosen as a future goal (*N* = 3, 6%). Almost all experts indicated administrative burden as a barrier (*N* = 48, 98%). Overall, experts endorsed facilitators more than the barriers for implementation (Fig. 2).

## Discussion

### Main findings

This three-round European Delphi study including 50 experts, resulted in a quality indicator set with 42 indicators with high-level of consensus on validity, feasibility, discriminability, and actionability, representing 13 clinical domains for patients with TBI at the ICU. Experts indicated multiple facilitators for implementation of the total set, while the main barrier was the anticipated administrative burden. The selection of indicators during the Delphi process gave insight in which quality indicators were perceived as important to improve the quality of TBI care. In addition, the indicator definitions evolved during the Delphi process, leading to a final set of understandable and easy to interpret indicators by (clinical) experts. This set serves as a starting point to gain insight into current ICU care for TBI patients, and after empirical validation, it may be used for quality measurement and improvement.

Our Delphi resulted in 17 structure indicators, 16 process indicators, and 9 outcome indicators. A large number of structure indicators already reached consensus after round 2; this might reflect that these were more concise indicators. However, during the rounds, definitions for process indicators became more precise and specific. Process indicators must be evidence-based before best practices can be determined: this might also explain that important domains with indicators on daily care in TBI (such as decompressive craniectomy, osmotic therapies, respiration, and ventilation management) did not reach consensus in our Delphi study. Structure, process, and outcome indicators have their own advantages and disadvantages. For example, process indicators tend to be inherently actionable as compared to structure and outcome indicators, yet outcome indicators are more relevant to patients [35]. Most indicators were excluded from the set due to low agreement and lack of consensus on actionability and validity: this indicates that experts highly valued the practicality and usability of the set and were strict on selecting only those indicators that might improve patient outcome and processes of care. Overall, the complete set comprises all different types of indicators.

### Existing indicators

Some national ICU registries already exist [17], and in 2012, a European ICU quality indicator set for general ICU quality has been developed [18]. In addition, several trauma databanks already exist [36, 37]. The motivations for selection (or rejection) of indicators in our study can contribute to the ongoing debate on which indicators to collect in these registries. For example, length of stay is often used as an outcome measure in current registries, but the Delphi panel commented that determination of the length of stay is debatable as an indicator, since hospital structures differ (e.g., step-down units are not standard), and admission length can be confounded by (ICU) bed availability. Although general ICU care is essential for TBI, not all general ICU or trauma indicators are applicable in exactly the same way for TBI. For example, individualized deep venous thrombosis prophylaxis management in TBI is a priority in view of the risk of progressive brain hemorrhage in contrast to other ICU conditions (e.g., sepsis). Therefore, our TBI-specific indicator set might form a useful addition to current registries.

### Strength and limitations

This study has several strengths and limitations. No firm rules exist on how to perform a Delphi study in order to develop quality indicators [19]. Therefore, we extensively discussed the methodology and determined strategies with the Advisory Committee. Although the RAND/UCLA Appropriateness Method recommends a panel meeting [38], no group discussion took place in our study to avoid overrepresentation of strong voices and for reasons of feasibility. However, experts received both qualitative and quantitative information on the rating of indicators to gain insight into the thinking process of the other panel members. Considering the preliminary indictor set, we used the guidelines [23, 24] as a guide to which topics should be included and not as an evidence base. Considering the Delphi panel, the success of indicator selection depends on the expertise of invited members: we assembled a large network of 50 experts from 18 countries across Europe with various professional backgrounds. All participants can be considered as established experts in the field of TBI-research and/or daily clinical practice (around 70% of experts had more than 10 years of ICU experience). However, more input from some key stakeholders in the quality of ICU care, such as rehabilitation physicians, nurses and allied health practitioners, health care auditors, and TBI patients, would have been preferable. We had only three rehabilitation experts on our panel, but increased input from this group of professionals would have been valuable, since they are increasingly involved in the care of patients even at the ICU stage. A number of nurses were invited, but none responded, possibly due to a low invitation rate. This is a severe limitation since nurses play a key role in ICU quality improvement and quality indicator implementation [39, 40]. Therefore, future studies should put even more efforts in involving nurses in quality indicator development. Experts were predominantly from Western Europe. Therefore, we performed sensitivity analyses for Western Europe and removed indicators with significant differences compared with other regions to obtain a set generalizable for Europe. The restriction to a European rather than a global set was motivated by substantial continental differences in health funding systems, health care costs, and health care facilities. Finally, some of the responses may have been strongly influenced by familiarity with measures (e.g., SF-36 was selected instead of Qolibri) rather that solely reflecting the value of the measure per se.

### Use and implementation

Quality indicators may be used for the improvement of care in several ways. First, registration of indicator data itself will make clinicians and other stakeholders aware of their center or ICU performance, as indicators will provide objective data on care instead of perceived care. Second, as the evidence base for guidelines is often limited, this indicator set could support refinement of guideline recommendations. This was shown in a study by Vavilala et al., where guideline-derived indicators for the acute care of children with TBI were collected from medical records and were associated with improved outcome [10]. Third, quality indicators can be used to guide and to inform quality improvement programs. One study showed that a TBI-specific quality improvement program was effective, demonstrating lower mortality rates after implementation [41]. Fourth, (international) benchmarking of quality indicators will facilitate discussion between (health care) professionals and direct attention towards suboptimal care processes [17]. Future benchmarking across different hospitals or countries requires advanced statistical analyses such as random effect regression models to correct for random variation and case-mix correction. To perform such benchmarking, case-mix variables must be collected, like in general ICU prognostic models or the TBI-specific prognostic models, such as IMPACT and CRASH [42, 43].

A quality indicator set is expected to be dynamic: ongoing large international studies will further shape the quality indicator set. This is also reflected in the “retirement” of indicators over time (when 90–100% adherence is reached). Registration and use of the quality indicators will provide increasing insight into their feasibility and discriminability and provides the opportunity to study their validity and actionability. Such empirical testing of the set will probably reveal that not all indicators meet the required criteria and thus will reduce the number of indicators in the set, which is desirable, as the set is still quite extensive. For now, based on the dynamic nature of the set and ongoing TBI studies, we recommend to use this consensus-based quality indicator for registry purposes—to gain insight (over time) in current care and not for changing treatment policies. Therefore, we recommend to regard this consensus-based quality indicator set as a starting point in need of further validation, before broad implementation can be recommended. Such validation should seek to establish whether adherence to the quality indicators is associated with better patient outcomes.

To provide feedback on clinical performance, new interventions are being explored to further increase the effectiveness of indicator-based performance feedback, e.g., direct electronic audit and feedback with suggested action plans [44]. A single (external) organization for data collection could enhance participation of multiple centers. International collaborations must be encouraged and further endorsement by scientific societies seems necessary before large-scale implementation is feasible. When large-scale implementation becomes global, there is an urgent need to develop quality indicators for low-income countries [36, 45]. An external organization for data collection could also reduce the administrative burden for clinicians. This is a critical issue, since administrative burden was indicated as the main barrier for implementation of the whole indicator set, although experts agreed on the feasibility of individual indicators. In the future, automatic data extraction might be the solution to overcome the administrative burden.

## Conclusion

This Delphi consensus study gives insight in which quality indicators have the potential to improve quality of TBI care at European ICUs. The proposed quality indicator set is recommended to be used across Europe for registry purposes to gain insight in current ICU practices and outcomes of patients with TBI. This indicator set may become an important tool to support benchmarking and quality improvement programs for patients with TBI in the future.

## Additional files

Additional file 1: Questionnaire round 1. (DOC 270 kb)

Additional file 2: Questionnaire round 2. (DOCX 79 kb)

Additional file 3: Questionnaire round 3. (DOCX 89 kb)

Additional file 4: Indicator selection and scores during the Delphi process. (DOCX 66 kb)

## Abbreviations

AC: Advisory Committee
CENTER-TBI study: Collaborative European NeuroTrauma Effectiveness Research in Traumatic Brain Injury study
CSR-R: Coma Recovery Scale - Revised
EHR: Electronic health records
GOSE: Glasgow Outcome Coma Scale Extended
ICP: Intracranial pressure
ICU: Intensive care unit
IQR: Interquartile range
Qolibri: Quality of life after brain injury
RPQ: Rivermead Post-Concussion Symptoms Questionnaire
SF-36: Short form health survey
TBI: Traumatic brain injury
