# Supplementary material for: Development of a quality indicator set to measure and improve quality of ICU care for patients with traumatic brain injury
Source: Crit Care. 2019 Mar 22;23:95. doi: 10.1186/s13054-019-2377-x (PMC6431034; doi:10.1186/s13054-019-2377-x)
Supplement: Supplementary file 1 — Questionnaire round 1. (DOC 270 kb) [file 13054_2019_2377_MOESM1_ESM.doc]

**Development of a clinical quality indicator set 1**

Development of a clinical quality indicator set for patients with Traumatic Brain Injury at the Intensive Care Unit

This survey is part of the prospective, longitudinal CENTER-TBI study. CENTER-TBI is a large European project that aims to improve the care for patients with traumatic brain injury www.center-tbi.eu

One of the aims of the CENTER-TBI study is to develop a clinical indicator set in order to improve the quality of care for patients with traumatic brain injury

The definition of quality indicators: 'Quality indicators are measurement tools, screens or flags that are used as guides to monitor, evaluate and improve the quality of patient care, clinical support services, and organizational functions that affect patient outcomes.' (Canadian Council on Health Services Accreditation:1996)

Quality indicators can be classified in structure, process, and outcome (Donabedian's framework). Structure indicators define the characteristics of the health system or the hospital in which the care is provided, such as human resources and organizational factors. Structure indicators are measured at the provider or system level. Process indicators can be measured per patient and refer to the appropriateness of the delivered care, such as guideline adherence. Outcome indicators reflect the end result as a consequence of care, such as clinical care outcomes, adverse events or patient's satisfaction with care. (Thesis quality indicators for hospital care, Claudia Fischer)

Please complete the questionnaire for adult patients with TBI in an ICU setting

At the end of the questionnaire you can comment on the used definitions of the quality indicators or give ideas for new indicators. The completion of the entire questionnaire will take about half an hour

You can save your answers and continue at a later moment. Please complete this questionnaire within 2 weeks.

There are 114 questions in this survey

# Agreement form

Thank you for taking the time to participate in this survey!

It is important that you understand that your participation is entirely voluntary. If you do not wish to take part in this study it will not influence your participation in the CENTER-TBI study. In addition, any information you provide is confidential. When the results of the data are reported you will not be identifiable in the findings.

**Name:**

Please write your answer here:

|  |
| --- |

**Please register with your email address:**

Please write your answer here:

|  |
| --- |

**Can we use your name in the additional file of the final paper (members of the Delphi panel)? Your name will not be linked to the results**

Please choose **only one** of the following:


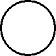
 Yes


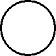
 No

**Gender:**

Please choose **only one** of the following:


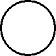
 Male


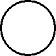
 Female

**Country:**

Please choose **only one** of the following:

|  |
| --- |

**What profession applies most to you?**

Please choose **only one** of the following:


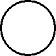
 neurosurgeon


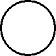
 intensivist


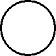
 ICU nurse


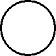
 neurologist


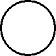
 anesthesiologist


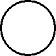
 trauma surgeon


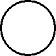
 ED physician


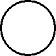
 rehabilitation specialist


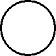
 methodologist/researcher/public health expert

Only complete the questionnaire if you are a specialists (residents are not invited to complete the Delphi)

**Role ICU (open question):**

|  |
| --- |

Please indicate your profession at the ICU in more detail, eg. neurointensivist or consultent ICU

**Are you the primary responsible/ in charge for the daily care of patient with TBI at the ICU?**

Please choose **only one** of the following:


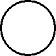
 No


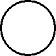
 Yes

**Number of years of professional experience at the ICU:**

**Only answer this question if the following conditions are met:**

Please choose **only one** of the following:


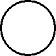
 3-5 years


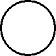
 5-10 years


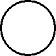
 10-15 years


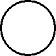
 more than 15 years

(also if you are consultant at the ICU)

**Number of years of professional experience in quality indicator research:**

Please choose **only one** of the following:


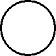
 3-5 years


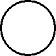
 5-10 years


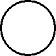
 10-15 years


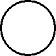
 more than 15 years

(also if you are consultant at the ICU)

**Your center is:**

Please choose **only one** of the following:


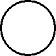
 Academic/ University


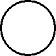
 Nonacademic

**What is the location of your center:**

Please choose **only one** of the following:


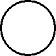
 urban location = a hopital in or very near to a city, the area is crowded


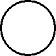
 suburban location = in between urban and rural location


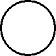
 rural location = a hospital in a location in or very near to the countryside, the area is not crowded

**Is your hospital officially designated as a trauma center:**

Please choose **only one** of the following:


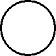
 Level 1


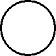
 Level 2


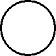
 Level 3


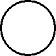
 Our center is not officially designated as a trauma center


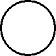
 *Our country* does not explicitly designate trauma centers

Level I trauma center: A regional resource center that generally serves large cities or population-dense areas. A level I trauma center is expected to manage large numbers of severely injured patients (at least 1,200 trauma patients annually or have 240 admissions with an Injury Severity Score of more than 14). It is characterized by 24-hour in-house availability of an attending surgeon and the prompt availability of other specialties (e.g. neurosurgeon, trauma surgeon).

Level II trauma center: A level II trauma center provides comprehensive trauma care in either a population-dense area in which a level II trauma center may supplement the clinical activity and expertise of a level I institution or occur in less population-dense areas. In the latter case, the level II trauma center serves as the lead trauma facility for a geographic area when a level I institution is not geographically close enough to do so. It is characterized by 24-hour in-house availability of an attending surgeon and the prompt availability of other specialties (e.g. neurosurgeon, trauma surgeon).

Level III trauma center: A level III trauma center has the capacity to initially manage the majority of injured patients and have transfer agreements with a level I or II trauma center for seriously injured patients whose needs exceed the facility’s resources.

**Do you have electronic patient records at your ICU:**

Please choose **only one** of the following:


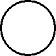
 Yes


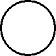
 No

**Are you participating in the CENTER-TBI study? (this does not affect your participation in the Delphi)**

Please choose **only one** of the following:


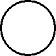
 Yes


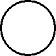
 No

# Instruction

**We developed indicators based on the BTF guidelines and the Provider Profilling questionnaires:**

- **Carney N. et al. Guidelines for the Management of Severe Traumatic Brain Injury,Fourth Edition, neurosurgery 2017**
- **Cnossen MC et al. Variation in monitoring and treatment policies for intracranialhypertension in traumatic brain injury: a survey in 66 neurotrauma center participating in the CENTER-TBI study. Critical Care 2017**
- **Huijben JA et al. Variation in general supportive and preventive intensive caremanagement of Traumatic Brain Injury: a survey in 66 neurotrauma centers participating in the Collaborative European NeuroTrauma Effectiveness Research in Traumatic Brain Injury (CENTER-TBI) study Critical Care (in press)**

**Please indicate if you think the indicators (numbered) can provide valid, feasible, disciminable and actionable information on the structures, processes and outcomes of adult patients with TBI at your ICU**

**Answer model (repeated for each proposed quality indicator)**

# Protocol

**Please indicate if you think the indicators (numbered) can provide valid, feasible, discriminable and actionable information on the structures, processes and outcomes of adult patients with TBI at your ICU**

**1. Structure: The existence of a protocol including specific guidelines (like the BTF**

**guidelines) for Traumatic Brain Injury patients (yes/no)**

Please choose the appropriate response for each item:

(5-point Likert scale):

|  | Strongly disagree | Disagree | Neither agree nor disagree | Agree | Strongly agree | I don’t know |
| --- | --- | --- | --- | --- | --- | --- |
| **Validity**: It is likely that better performance on the indicator reflects better processes of care and leads to better patient outcome | 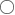 | 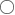 | 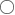 | 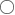 | 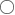 | 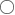 |
| **Discriminability**: It is expected that there is variability in clinical practice | 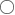 | 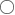 | 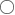 | 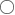 | 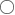 | 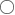 |
| **Feasibility**: Measurement of the indicator is feasible  (data for the indicator are available or easy to obtain) | 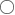 | 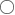 | 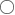 | 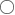 | 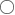 | 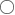 |
| **Actionability**: The indicator can be used to improve quality of care | 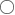 | 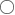 | 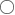 | 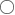 | 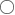 | 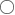 |

**2. Structure: The presence of (some form of) regular audits to check guideline adherence in your Intensive Care Unit (ICU) (yes/no)**

## 3. Structure: The presence of dedicated persons(s) to oversee guidelines development and maintenance for patients with TBI at the ICU (yes/no)

**Addition of quality indicators or comments for this group? (Repeated at the end of each topic)**

**/Ideas for clearer definitions:**

Please write your answer here:

please keep in mind that we would like to develop an uniform and minimal European clinical indicator set

# Intensive Care Unit

**4. Structure: Does your hospital have a dedicated/specialized neurocritical care unit?**

**(yes/no)**

**5. Number of patients with ICP monitoring/ number of patients with indication for ICP monitoring**

Based on the BTF guidelines: indication for ICP monitoring: all salvageable patients with TBI and GCS 3-8 after resuscitation and an abnormal CT scan OR severe TBI patients with a normal CT and 2 of more of the following 1) age > 40 years, 2) unilateral or bilateral motor posturing (= M2 or M3), 3) SBP>90 mmHg

**6. Structure: The availability of operating rooms 24 hours per day (yes/no)**

**7. Structure: Number of patients with TBI treated in your ICU annually**

**8. Structure: Annual number of severe TBI patients GCS<8 on admission admitted to the ICU (volume)**

**9. Structure: The presence of a step down unit where patients can still be monitored24/7, but less intensively than at the ICU (yes/no)**

A facility in-between ICU and ward. It is often used for patients who improved at the intensive care and no longer need the intensity of ICU care, but are also not well enough to be cared for at the ward. The care provided in step down beds is less intensive than the care provided at the ICU but more intensive than ward care

# Staff

**10. Structure: Certified intensivist present in person 7 days a week during at least day-**

**time (yes/no)**

**11. Structure: Availability of a neurosurgeon (staff) 24/7 within 30 minutes after call**

**(yes/no)**

**12. Structure: Intensivist to ICU bed ratio**

**13. ICU nurse to bed ratio**

# CT scan

**14. Structure: 24/7 availability of a CT scan (yes/no)**

**15. Structure: Number of routinely repetitive CT scanning in patients with severe TBI considered at high risk for deterioration (abnormal CT and/ or ICP monitoring at ICU) / number of patients with severe TBI considered at high risk for deterioration (abnormal CT and/or ICP monitoring at ICU)**

Routine repeat CT scan refers to CT scans that are scheduled at the beginning of ICU admission, and undertaken at the predetermined time points regardless of clinical situation

# ICP-monitoring

1. **Structure: 24/7 availability of a certified person at your center that can insert an ICP monitor within 2 hours after admission at the ICU (yes/no)**
2. **Structure: Is the ICP monitor is zerood at the foramen of Monro in your clinic according to a protocol? yes/no**

**18. Outcome: Number of EVD infections/ total number of patients with TBI at the ICU with an EVD inserted**

# Precautions ICP-monitoring

1. **Process: Antibiotics are given prior to ICP monitor insertion/ number of patients**

**with TBI at ICU with ICP monitor**

1. **Process: Number of patients that have a coagulation panel assessed prior to insertion of an ICP monitor/ number of patients with TBI at the ICU and ICP monitor**

# Sedatives

**21. Process: Number of patients with high dose barbiturate administration/ patients with refractory ICP in spite of maximum standard institutional medical (osmotic) and surgical (space occupying lesions extracted on admission) treatment**

# Osmotic therapies

**22. Process: Number of patients with TBI receiving mannitol (at doses of 0.25 to 1 g/kg**

**body weight)/number of TBI patients receiving mannitol**

**23. Outcome: Number of TBI patients with a positive fluid balance > 1 L or negative fluid balance >-0.5 L (at day 1, 2 or 3) / number of patients with TBI at ICU**

# Seizures

**24. Indicator: Number of patients with TBI receiving antiepileptic medication to prevent early seizures/ total number of patients with TBI at the ICU**

# Fever

**25. Structure: Is there 24/7 availability of cooling devices to perform targeted temperature management for each patient with TBI at the ICU? (yes/no)**

# DVT

**26. Process: Number of patients with TBI that receive mechanical DVT prophylaxis (e.g. stockings)/ total number of patients with TBI at the ICU**

DVT: deep venous thrombosis

## 27. Process: Number of patients with TBI at the ICU that receive medical prophylaxis with low molecular weight heparins / total number of TBI patients admitted to the ICU with (any) blood on CT

**28. Process: Number of patients with TBI that receive medical prophylaxis with (low molecular weight) heparins within the first *72 hours* after admission/ total number of TBI patients admitted to the ICU with (any) blood on CT**

DVT: deep venous thrombosis

**29. Outcome: Number of TBI patients with confirmed deep venous thrombosis or pulmonary embolism /total number of TBI patients at the ICU**

**30. Outcome: Number of patients with cerebral hemorrhage (any blood) on early CT/ total number of TBI patients at the ICU**

Progressive hemorrhagic injury: presence of new intracerebral hematomas, coalescence of pre-existing contusions into a hematoma, delayed or enlargement of subdural or epidural hematomas, or development of unexpected postsurgical hematomas at the operative site (Vedentam et al. J Neurosurg. 2016)

# Intermediate questions

**Quality indicators provide useful information (on daily performance of TBI care in my center)**

Please choose **only one** of the following:


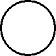
 Yes


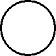
 No

**Should the target population be isolated traumatic brain injury patients (instead of polytrauma patients)?**

Please choose **only one** of the following:


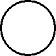
 Yes


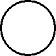
 No

**Should we include patient reported outcome measures (PROMs) in the indicator set for TBI at the ICU?**

Please choose **only one** of the following:


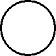
 Yes


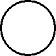
 No

A patient reported outcome is directly reported by the patient without interpretation of the patient's response by a clinician or anyone else and pertains to the patient's health, quality of life, or functional status associated with health care or treatment

PROMs are the tools or instruments to measure patient reported outcomes

**(Due to the limited evidence base for processes in TBI) an indicator set should primarily focus on outcomes, do you agree?**

Please choose **only one** of the following:


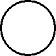
 Yes


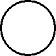
 No


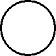
 I don't know

**Should the indicator set be part of a larger registry like a global trauma registry?**

Please choose **only one** of the following:


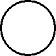
 Yes


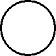
 No


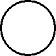
 I don't know

**Are we ready for benchmarking ('the process of identifying and learning from good practices in other organizations') or should we start with a registry (gain information) in the coming 5 years?**

Please choose **only one** of the following:


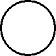
 Yes


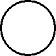
 No benchmarking

Benchmarking defined by European Benchmarking Code of conduct

**Should pay for performance (reward high quality or pay for below standard quality) exist for TBI care in the coming 5 years?**

Please choose **only one** of the following:


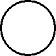
 Yes


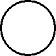
 No

# Coagulopathy

**30. Structure: At your ICU is viscoelastic testing available for TBI patients? yes/no**

**31. Outcome: Number of patients with TBI with platelets below 75 x 10^9^ /L at least in one lab results/ total number of patients with TBI at ICU**

# Respiration and ventilation

**33. Process: Number of TBI patients with a tracheostomy within 2 weeks after admission to the ICU/ number of patients with a tracheostomy placed during ICU stay**

| **34. Process: Number of mechanical ventilated patients with TBI administered analgetics/ number of mechanical ventilated patients with TBI at the ICU** |  |
| --- | --- |

**35. Outcome: Number of patients (in the absence of cerebral herniation) with partial pressure of carbon dioxide in arterial blood (PaCO2) outside normal range at any time / TBI patient-days at the ICU**

# Red blood cell policy

**36. Outcome: Number of patients with too low Hb (compared to protocol target level) at**

**any time during ICU stay / total number of patients with TBI at ICU**

**37. Outcome: Number of TBI patients with an arterial base deficit > 2 mmol/L below or above upper normal range limit within 48 hours after admission/ total number of TBI**

**patients at the ICU**

# Glucose

**38. Structure: Do you have a protocol for glucose management available for patients**

**with TBI at your ICU? yes/no**

**39. Outcome: Number of lab results in patients with TBI with a blood glucose above the**

**target level in your protocol/ number of TBI patients at the ICU**

# Nutrition

**40. Process: Number of TBI patients with basal full caloric replacement within 5 to 7**

**days post-injury / number of TBI patients at the ICU**

**41. Process: Number of TBI patients with transgastric jejenual feeding/ number of**

**patients with TBI at ICU**

**42. Process: Number of TBI patients with basal full caloric replacement within 72 hours/ number of TBI patients at the ICU**

# Surgery

**43. Structure: The presence of a protocol/ institutional guideline that provides**

**indications for surgery with SDH and EDH (yes/no)**

SDH: subdural hematoma EDH: epidural hematoma

**44. Process: Number of decompressive craniectomies/ number of patients with TBI with ICP refractory to maximum treatment dose with osmotic agents according to institutional guidelines**

Refractory high ICP: high ICP refractory to conventional first-tier therapies (CSF removal, mannitol, sedation, paralysis, mild hyperventilation)

**45. Process: Number of large frontotemporoparietal decompressive craniectomies (not less than 12 x 15 cm or 15 cm diameter) / number of patients with decompressive craniecomies**

Refractory high ICP: high ICP refractory to conventional first-tier therapies (CSF removal, mannitol, sedation, paralysis, mild hyperventilation)

# Paramedics

## 46. Structure: A structural weekly meeting between intensivist and neurosurgeon to discuss TBI patients at the ICU (yes/no)

**47. Process: Number of patients with TBI with prevention of contractures during ICU stay (using passive movements)/ total number of patients with TBI at the ICU admitted at least 2 weeks**

**48. Process: Number of patients with TBI visited by a physiotherapist during ICU stay/**

**total number of patients with TBI at the ICU *admitted at least 7 days***

**49. Process: Number of patients with a rehabilitation plan after ICU discharge/ number**

**of patients discharged from ICU**

# Assessment scales at the ICU

**50. Structure: Additional brain targeted monitoring (next to ICP/CPP and CO2) like PBrO2, SjO2, metabolic brain monitoring, NIRS, TCD etc/ (yes/no, how much modalities)**

PbrO_2_: partial pressure of brain tissue oxygen

SjO_2_: oxygen saturation of jugular bulb

NIRS: near- infrared spectroscopy

TCD: transcranial Doppler

**51. Process: Number of TBI patients with daily assessment of the Glasgow Coma Scale(GCS)/ number of TBI patients at the ICU**

**52. Process: Number of assessments of delirium presence with validated screening tool/ total number of ICU days in TBI patients**

**53. Process: Daily visit by neurosurgeon/ TBI patientdays at ICU**

**54. Process: Information on prognosis discussed with family by one of the treating physicians (ICU phyician or neurosurgical physician) at least once/ total number of TBI patients at ICU**

# Short term outcomes

**55. Process: Number of patients with TBI with a structural interview Glasgow outcome**

**scale (extended) at hospital discharge/ number of discharged patients**

**56. Process: Number of patients with TBI with a structural interview Glasgow outcome scale (extended) at follow-up (at least after 3 months)/ number of discharged patients**

**57. Process: Number of patients with TBI discharged to a rehabilitation center/ total**

**number of patients with TBI admitted to the ICU**

**58. Outcome: The mean overall length of stay in the hospital of TBI patients**

**59. Outcome: The mean overall length of stay in the ICU of TBI patients**

**60. Outcome: Number of in-hospital deaths among patients with TBI/ total number of admitted patients with TBI**

**61. Outcome: Incidence of ventilator associate pneumonia (VAP) in patients with TBI/ total number of TBI patients with mechanical ventilation**

**62. Outcome: Number of patients with TBI with severe sepsis or septic shock/ total**

**number of patients with TBI at the ICU**

Sepsis should be defined as life-threatening organ dysfunction caused by a dysregulated host response to infection. For clinical operationalization, organ dysfunction can be represented by an increase in the Sequential [Sepsis-related] Organ Failure Assessment (SOFA) score of 2 points or more, which is associated with an in-hospital mortality greater than 10%. Septic shock should be defined as a subset of sepsis in which particularly profound circulatory, cellular, and metabolic abnormalities are associated with a greater risk of mortality than with sepsis alone. Patients with septic shock can be clinically identified by a vasopressor requirement to maintain a mean arterial pressure of 65 mm Hg or greater and serum lactate level greater than 2 mmol/L (>18 mg/dL) in the absence of hypovolemia.

Singer et al. The Third International Consensus Definitions for Sepsis and Septic Shock (Sepsis-3) JAMA 2016

# Long term outcomes

**63. Process: Number of patients with TBI receiving follow-up by a specialist within 2**

**months after discharge/ total number of patients with TBI discharged**

**64. Outcome: The rate of brain complications at.. months**

**When should the outcome indictor (brain complications) be measured?**

Please choose **only one** of the following:


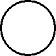
 3 months


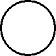
 6 months


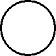
 12 months


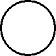
 24 months


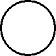
 3, 6, 12 months
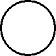
 6, 12 months


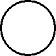
 at all time points

**65. Outcome: The rate of systemic complications at.. months**

**When should the outcome indicator (systemic complications) be measured?**

Please choose **only one** of the following:


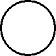
 3 months


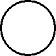
 6 months


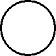
 12 months


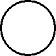
 24 months


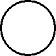
 3, 6, 12 months
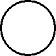
 6, 12 months


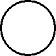
 at all time points

**66. Outcome: Total number of patients that returned to work/school at .. months**

**When should the outcome indicator (return to work/school) be measured?**

Please choose **only one** of the following:


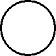
 3 months


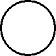
 6 months


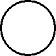
 12 months


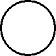
 24 months


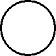
 3, 6, 12 months
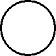
 6, 12 months


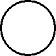
 at all time points

# Outcome scales

**Please rate the following outcome scales that you believe are most important and feasible to include in an outcome indicator set (for example, the number of patients with a certain GOSE score can be compared between hospitals)**

All your answers must be different and you must rank in order.

Please select between 1 and 8 answers

Please number each box in order of preference from 1 to 24 Please choose at least 1 items.

Please choose no more than 8 items.

GOSE (glasgow outcome scale extended)

CRS-R (coma recovery scale revised)

RPQ (Rivermead Post-Concussion Symptoms Questionnaire)

SF-12 (short form health survey 12)

SF-36 (short form health survey 36)

EQ-5D

QoliBRI (quality of life after brain injury)

WAIS Processing Speed Index

Cog-FIM (cognitive Functional Independence Measure)

FIM motor subscale (Functional Independence Measure)

CHART-SF (The Craig Handicap Assessment and Reporting Technique Short Form)

SWLS (Satisfaction With Life Scale)

PCL-5 (post-traumatic stress disorder)

Rivermead post-concussion questionnaire

BSI-18 Brief Symptom Inventory

PHQ-9 (depression patient health questionnaire)

GAD-7 (Anxiety test questionnaire)

GOAT (Galveston Orientation and Amnesia Test)

TMT (trial making test)

RAVLT (rey auditory and verbal learning test)

10 m walk test

Timed up and go

CANTAB (cognitive Research software)

I don’t know

Submit your survey.

Thank you for completing this survey.
