# Supplementary material for: Development of a quality indicator set to measure and improve quality of ICU care for patients with traumatic brain injury
Source: Crit Care. 2019 Mar 22;23:95. doi: 10.1186/s13054-019-2377-x (PMC6431034; doi:10.1186/s13054-019-2377-x)
Supplement: Supplementary file 2 — Questionnaire round 2. (DOCX 79 kb) [file 13054_2019_2377_MOESM2_ESM.docx]

**Development of a clinical quality indicator set**

Development of a clinical quality indicator set for patients with Traumatic Brain Injury at the Intensive Care Unit: a Delphi consensus study

This Delphi study is part of the prospective, longitudinal CENTER-TBI study. CENTER-TBI is a large European project that aims to improve the care for patients with traumatic brain injury ([www.center-tbi.eu](http://www.center-tbi.eu/))

One of the aims of the CENTER-TBI study is to develop a clinical indicator set in order to improve the quality of care for patients with traumatic brain injury

Quality indicators are defined as follows: 'Quality indicators are measurement tools, screens or flags that are used as guides to monitor, evaluate and improve the quality of patient care, clinical support services, and organizational functions that affect patient outcomes.' (Canadian Council on Health Services Accreditation:1996)

Quality indicators can be classified in structure, process, and outcome (Donabedian's framework). Structure indicators define the characteristics of the health system or the hospital in which the care is provided, such as human resources and organizational factors. Structure indicators are measured at the provider or system level. Process indicators can be measured per patient and refer to the delivered care, such as guideline adherence. Outcome indicators reflect the end result as a consequence of care, such as clinical care outcomes and adverse events. (Thesis quality indicators for hospital care, Claudia Fischer)

Please complete the questionnaire for adult patients with TBI in an ICU setting

For each topic of the questionnaire you can comment on the used definitions of the quality indicators or give ideas for new indicators. The completion of the entire questionnaire will take about half an hour

You can save your answers and continue at a later moment. Please complete this questionnaire within 2 weeks.

There are 84 questions in this survey

# Agreement form

Thank you for taking the time to participate in this survey!

It is important that you understand that your participation is entirely voluntary. If you do not wish to take part in this study it will not influence your participation in the CENTER-TBI study. In addition, any information you provide is confidential. When the results of the study are reported, you will not be identifiable in the findings.

## Name:

Please write your answer here:

## Please register with your email address:

Please write your answer here:

## Can we use your name in the additional file of the final paper (members of the Delphi panel)? Your name will not be linked to the results

Please choose **only one** of the following:


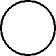
 Yes
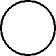
 No

## Gender:

Please choose **only one** of the following:


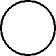
 Male


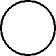
 Female

## Country:

Please choose **only one** of the following:

|  |
| --- |

## What profession applies most to you?

Please choose **only one** of the following:


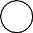
 neurosurgeon


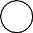
 intensivist


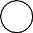
 ICU nurse


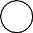
 neurologist


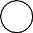
 anesthesiologist


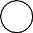
 trauma surgeon


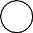
 ED physician


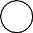
 rehabilitation specialist


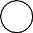
 methodologist/researcher/public health expert


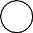
 other

Only complete the questionnaire if you are a specialists (residents are not invited to complete the Delphi)

## Role ICU (open question):

|  |
| --- |

Indicate your profession at the ICU in more detail, eg. neurointensivist or consultent ICU

## Are you the primary responsible/ in charge for the daily care of patient with TBI at the ICU?

Please choose **only one** of the following:


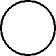
 Yes
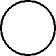
 No

## Number of years of professional experience at the ICU (or with TBI patients at another

**department):**

Please choose **only one** of the following:


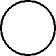
 3-5 years


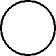
 5-10 years


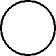
 10-15 years


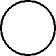
 more than 15 years

(also if you are consultant at the ICU)

## Number of years of professional experience in quality indicator research:

Please choose **only one** of the following:


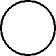
 3-5 years


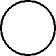
 5-10 years


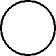
 10-15 years


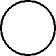
 more than 15 years

## Your center is:

Please choose **only one** of the following:


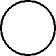
 Academic/ University


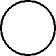
 Nonacademic

## What is the location of your center:

Please choose **only one** of the following:


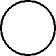
 urban location = a hopital in or very near to a city, the area is crowded


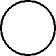
 suburban location = in between urban and rural location


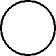
 rural location = a hospital in a location in or very near to the countryside, the area is not crowded

## Is your hospital officially designated as a trauma center:

Please choose **only one** of the following:


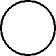
 Level 1


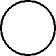
 Level 2


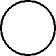
 Level 3


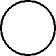
 Our center is not officially designated as a trauma center
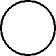
 Our country does not explicitly designate trauma centers

Level I trauma center: A regional resource center that generally serves large cities or population-dense areas. A level I trauma center is expected to manage large numbers of severely injured patients (at least 1,200 trauma patients annually or have 240 admissions with an Injury Severity Score of more than 14). It is characterized by 24-hour in-house availability of an attending surgeon and the prompt availability of other specialties (e.g. neurosurgeon, trauma surgeon).

Level II trauma center: A level II trauma center provides comprehensive trauma care in either a population-dense area in which a level II trauma center may supplement the clinical activity and expertise of a level I institution or occur in less population-dense areas. In the latter case, the level II trauma center serves as the lead trauma facility for a geographic area when a level I institution is not geographically close enough to do so. It is characterized by 24-hour in-house availability of an attending surgeon and the prompt availability of other specialties (e.g. neurosurgeon, trauma surgeon).

Level III trauma center: A level III trauma center has the capacity to initially manage the majority of injured patients and have transfer agreements with a level I or II trauma center for seriously injured patients whose needs exceed the facility’s resources.

## Do you have electronic patient records at your ICU:

Please choose **only one** of the following:


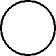
 Yes


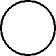
 No

## Are you participating in the CENTER-TBI study? (this does not affect your participation in the Delphi)

Please choose **only one** of the following:


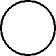
 Yes


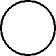
 No

# Instruction

## We developed indicators based on the BTF guidelines and the Provider Profiling questionnaires:

- **Carney N. et al. Guidelines for the Management of Severe Traumatic Brain Injury, Fourth Edition, neurosurgery 2017**
- **Cnossen MC et al. Variation in monitoring and treatment policies for intracranial hypertension in traumatic brain injury: a survey in 66 neurotrauma center participating in the CENTER-TBI study. Critical Care 2017**
- **Huijben JA et al. Variation in general supportive and preventive intensive care management of Traumatic Brain Injury: a survey in 66 neurotrauma centers participating in the Collaborative European NeuroTrauma Effectiveness Research in Traumatic Brain Injury (CENTER-TBI) study Critical Care 2018**

**Please indicate if you think the indicators can provide valid, discriminable, feasible, and actionable information on the structures, processes and outcomes of adult patients with TBI at your ICU**

**Answer model (repeated for each proposed quality indicator)**

**Protocol**

Please indicate if you think the indicators (numbered) can provide valid, discriminable, feasible, and actionable information on the structures, processes and outcomes of adult patients with TBI at your ICU

## 1.Structure: The existence of a protocol including specific guidelines (like the BTF guidelines or institutional guidelines) for Traumatic Brain Injury patients (yes/no)

Please choose the appropriate response for each item:

(5-point Likert scale):

|  | Strongly disagree | Disagree | Neither agree nor disagree | Agree | Strongly agree | I don’t know |
| --- | --- | --- | --- | --- | --- | --- |
| **Validity**: It is likely that better performance on the indicator reflects better processes of care and leads to better patient outcome | 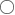 | 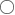 | 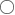 | 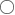 | 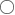 | 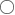 |
| **Discriminability**: It is expected that there is variability in clinical practice | 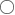 | 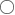 | 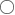 | 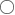 | 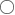 | 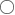 |
| **Feasibility**: Measurement of the indicator is feasible  (data for the indicator are available or easy to obtain) | 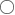 | 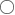 | 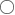 | 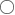 | 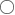 | 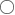 |
| **Actionability**: The indicator can be used to improve quality of care | 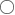 | 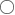 | 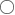 | 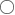 | 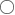 | 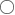 |

## 2.Structure: The presence of (some form of) regular audits to check guideline adherence in general at the Intensive Care Unit (ICU) (yes/no)

Audits do not have to be specific for TBI

## 3.Structure: The presence of dedicated person(s) to oversee guidelines development and maintenance, including those for patients with TBI, at the ICU (yes/no)

## Addition of quality indicators or comments for this group? (repeated at the end of each topic)

**/Ideas for clearer definitions:**

Please write your answer here:

# Intensive Care Unit

## 4. Structure: Does your hospital have a dedicated/specialized neurocritical care unit? (yes/no)

## 5. Structure: The availability of operating rooms 24 hours per day (yes/no)

## 6. Structure: The presence of a step down unit where patients can still be monitored 24/7, but less intensively than at the ICU (yes/no)

A facility in-between ICU and ward. It is often used for patients who improved at the intensive care and no longer need the intensity of ICU care, but are also not well enough to be cared for at the ward. The care provided in step down beds is less intensive than the care provided at the ICU but more intensive than ward care

# Staff

## 7. Structure: Certified intensivist present in person 7 days a week during at least day- time (yes/no)

## 8. Structure: Availability of a neurosurgeon (staff) 24/7 within 30 minutes after call (yes/no)

## 9. Structure: Intensivist to ICU bed ratio

## 10. Structure: ICU nurse to bed ratio

## 11. Structure: A structural meeting 2-3 times a week between intensivist and neurosurgeon to discuss TBI patients at the ICU (yes/no)

## 12. Process: Number of daily visits of TBI patients by a neurosurgeon/ total number of ICU days in TBI patients

# CT scan

## 13. Structure: 24/7 availability of a CT scan (yes/no)

# ICP-monitoring

## 14. Structure: Number of severe TBI patients with ICP monitoring/ number of severe

## TBI patients at the ICU

GCS 3-8

## 15. Structure: 24/7 availability of a certified person at your center that can insert an ICP monitor within 2 hours after admission at the ICU (yes/no)

## 16. Structure: Is the ventricular catheter zeroed at the foramen of Monro according to a protocol? (yes/no)

## 17. Outcome: Number of EVD infections in patiens with TBI/ total number of patients with TBI at the ICU with an EVD inserted

Only for centers that use ventricular catheters

# Osmotic therapies

## 18. Process: Number of TBI patients with osmotic therapies employed maximally (plasma osmolality 320 mosm/l or Na up to 160 mmol/l) before resorting to 3rd tier therapies (barbiturates, decompressive craniectomy) / number of TBI patients with 3rd tier therapies (barbiturates, decompressive craniectomy)

# Deep Venous Thrombosis (DVT)

## 19. Process: Number of patients with TBI that receive DVT prophylaxis /total number of patients with TBI at the ICU

Extra: Timing (days from the injury) and type of DVT prophylaxis (mechanical and/or pharmaceutical) can be registered

## 20. Process: Number of patients with TBI that receive mechanical DVT prophylaxis (e.g. stockings) initiated within 6 hours/ total number of patients with TBI at the ICU

## 21. Process: Number of patients with TBI at the ICU that receive pharmaceutical prophylaxis with low molecular weight heparins / total number of TBI patients admitted to the ICU

This QI is about the choice of prophylaxis (low molecular weight heparin), not about timing

## 22. Outcome: Number of TBI patients with confirmed DVT / total number of TBI patients at the ICU

This QI will be adjusted for case-mix and mortality data and is only applicable in centers that routinely perform ultrasound of the legs

# Coagulopathy

# 23. Structure: At your ICU is viscoelastic testing available for TBI patients? yes/no

# Respiration and ventilation

## 24. Process: Number of TBI patients with a tracheostomy within 2 weeks after admission to the ICU/ number of TBI patients at the ICU

## 25. Process: Number of mechanical ventilated patients with TBI administered opioids/ number of mechanical ventilated patients with TBI at the ICU

## 26. Outcome: Number of TBI patients with the presence of abnormally low PaCO2 (<4 kPa) in the absence of intracranial hypertension/ number of patients with TBI at the ICU

This QI will be adjusted for case-mix and mortality data

# Glucose

## 27. Structure: Do you have a protocol for glucose management available for patients with TBI at your ICU? yes/no

## 28. Outcome: Number of TBI patients with any blood glucose below 4 mmol/L (hypoglycemia)/ number of TBI patients at the ICU

# Nutrition

## 29. Process: Number of TBI patients with basal full caloric replacement within 5 to 7 days post-injury / number of TBI patients at the ICU

## 30. Process: Number of TBI patients with transgastric jejenual feeding to reduce ventilator associated pneumonia within 7 days after ICU admission/ number of patients with TBI at ICU

# Surgery

## 31. Structure: The presence of a protocol or institutional guideline that provides indications for surgery with SDH and EDH (yes/no)

SDH: subdural hematoma EDH: epidural hematoma

## 32. Process: Number of decompressive craniectomies in TBI patients/ number of patients with TBI with ICP refractory to maximum treatment dose with osmotic agents according to institutional guidelines

Refractory high ICP: high ICP refractory to conventional first-tier therapies (CSF removal, mannitol, sedation, paralysis, mild hyperventilation)

# Paramedics

## 33. Outcome: Number of major contractures (hips, knee, ankle, shoulder, elbow, wrist) in TBI patients/ total number of patients with TBI at the ICU

This QI wil be adjusted for case-mix and mortality data. Contracture is defined as the maximum passive range of motion of less than neutral position (also neutral body position: e.g. for the ankle with knee in extension)

## 34. Process: Number of patients with TBI visited daily by a physiotherapist during ICU stay/ total number of patients with TBI at the ICU

## 35. Process: Number of TBI patients with a rehabilitation plan after ICU discharge/ number of TBI patients discharged from the ICU

# Assessment scales at the ICU

## 36. Process: Number of daily assessments (3x or more) of the Glasgow Coma Scale (GCS) in TBI patients/ total number of ICU days in TBI patients

## 37. Process: Number of assessments of delirium presence with validated screening tool in conscious TBI patients/ total number of ICU days in conscious TBI patients

## 38. Structure: Information on prognosis is discussed with family by one of the treating physicians (ICU phyician or neurosurgical physician) at least once during ICU stay

# Short term outcomes

# 39. Outcome: The median overall length of stay in the hospital of TBI patients

## This QI will be adjusted for case-mix and mortality data

## 40. Outcome: The median overall length of stay in the ICU of TBI patients

## This QI will be adjusted for case-mix and mortality data

## 41. Outcome: Number of in-hospital deaths among patients with TBI/ total number of admitted patients with TBI

This QI will be adjusted for case-mix and mortality rates

## 42. Outcome: Incidence of ventilator associated pneumonia (VAP) in patients with TBI/ total number of TBI patients with mechanical ventilation at the ICU

This QI will be adjusted for case-mix and mortality rates

Pneumonia defined as 'the presence of new lung infiltrate plus clinical evidence that the infiltrate is of an infectious origin, which includes the new onset of fever, purulent sputum, leukocytosis, and decline in oxygenation.'

VAP is defined as a pneumonia occurring >48 hours after endotracheal intubation

*American Thoracic Society; Infectious Diseases Society of America. Guidelines for the management of adults with hospital- acquired, ventilator-associated, and healthcare-associated pneumonia. Am J Respir Crit Care Med. 2005*

**43. Outcome: Number of patients with TBI with severe sepsis or septic shock/ total number of patients with TBI at the ICU**

This QI will be adjusted for case-mix and mortality rates

Sepsis should be defined as life-threatening organ dysfunction caused by a dysregulated host response to infection. For clinical operationalization, organ dysfunction can be represented by an increase in the Sequential [Sepsis-related] Organ Failure Assessment (SOFA) score of 2 points or more, which is associated with an in-hospital mortality greater than 10%. Septic shock should be defined as a subset of sepsis in which particularly profound circulatory, cellular, and metabolic abnormalities are associated with a greater risk of mortality than with sepsis alone. Patients with septic shock can be clinically identified by a vasopressor requirement to maintain a mean arterial pressure of 65 mm Hg or greater and serum lactate level greater than 2 mmol/L (>18 mg/dL) in the absence of hypovolemia.

*Singer et al. The Third International Consensus Definitions for Sepsis and Septic Shock (Sepsis-3) JAMA 2016*

# Long term outcomes

## 44. Process: Number of patients with TBI with a structural interview to assess the Glasgow Outcome Scale (extended) at follow-up (at least after 3 months)/ number of discharged patients with TBI and ICU stay who survived up to 3 months

## 45. Process: Number of patients with TBI receiving follow-up by a specialist within 2 months after discharge/ total number of patients with TBI discharged (not in a rehab clinic)

## 46. Outcome: Total number of TBI patients that returned to work/school/ household activities at .. months/ number of TBI patients alive at ... months

## When should the outcome indicator (return to work/school/household activities) be measured?

Please choose **only one** of the following:


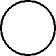
 3 months


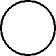
 6 months


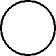
 12 months


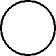
 24 months


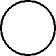
 3, 6 months


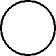
 6, 12 months


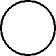
 3, 6, 12 months


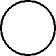
 12, 24 months
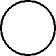
 at all time points

**Outcome scales**

**Please rate the following outcome scales that you believe are most important and feasible to include in an outcome indicator set (for example, the number of patients with a certain GOSE score can be compared between hospitals)**

All your answers must be different and you must rank in order.

Please select between 1 and 8 answers

Order of preference

GOSE (glasgow outcome scale extended)

CRS-R (coma recovery scale revised)

RPQ (Rivermead Post-Concussion Symptoms Questionnaire)

SF-12 (short form health survey 12)

SF-36 (short form health survey 36)

EQ-5D

QoliBRI (quality of life after brain injury)

WAIS Processing Speed Index

Cog-FIM (cognitive Functional Independence Measure)

FIM motor subscale (Functional Independence Measure)

CHART-SF (The Craig Handicap Assessment and Reporting Technique Short Form)

SWLS (Satisfaction With Life Scale)

PCL-5 (post-traumatic stress disorder)

Rivermead post-concussion questionnaire

BSI-18 Brief Symptom Inventory

PHQ-9 (depression patient health questionnaire)

GAD-7 (Anxiety test questionnaire)

GOAT (Galveston Orientation and Amnesia Test)

TMT (trial making test)

RAVLT (rey auditory and verbal learning test)

10 m walk test

Timed up and go

CANTAB (cognitive Research software)

I don’t know

## When should the outcome scales be measured?

Please choose **only one** of the following:


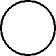
 3 months


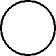
 6 months


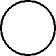
 12 months


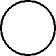
 24 months


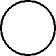
 3, 6 months


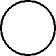
 6, 12 months


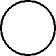
 3, 6, 12 months


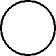
 12, 24 months
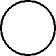
 at all time points

# Final questions

## The clinical quality indicator set (for TBI patients at the ICU) should be used for (multiple options):

Please choose **all** that apply:


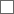
 Registry purposes (providing insight with the QI data)
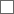
 Assessment of adherence to the guidelines


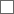
 Internal benchmarking (within a center)
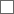
 External benchmarking (between centers)
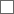
 Quality improvement programs


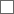
 Pay for performance


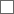
 None of the above: I do not believe an indicator set should be used at all
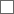
 Other:

## I do not believe an indicator set should be used at all, because

Please write your answer here:

## Do you have ideas for quality indicators on different topics? Do you have additional comments?

Submit your survey.

Thank you for completing this survey.
