# Supplementary material for: Development of a quality indicator set to measure and improve quality of ICU care for patients with traumatic brain injury
Source: Crit Care. 2019 Mar 22;23:95. doi: 10.1186/s13054-019-2377-x (PMC6431034; doi:10.1186/s13054-019-2377-x)
Supplement: Supplementary file 3 — Questionnaire round 3. (DOCX 89 kb) [file 13054_2019_2377_MOESM3_ESM.docx]

**Final Delphi round**

Development of a clinical quality indicator set for adult patients with Traumatic Brain Injury at the Intensive Care Unit

This is the final questionnaire of this Delphi study. We would like to thank you again for your participation.

In this final Delphi round we included quality indicators that did not achieve consensus yet (due to the scoring or comments). In addition, we included new indicator definitions that were proposed. The comments from the previous round can be found below the indicators. Sometimes we adjusted definitions based on these comments, sometimes we, after extensive discussion, decided not to incorporate the comments.

Please (re)rate the indicators in this questionnaire

The completion of the entire questionnaire will take about 20 minutes (less time than previous questionnaire)

You can save your answers and continue at a later moment. Please complete this questionnaire within 2 weeks.

There are 44 questions in this survey

# Collaborator

## Would you like to be listed as collaborator of this Delphi study? This means that we will facilitate academic credits as a collaborator for this study, meaning that you will be listed as collaborator for this study in PubMed online. If you do, please provide your full name and academic affiliation in this textbox:

Please write your answer here:

**Answer model (repeated for each proposed quality indicator)**

# Intensive Care Unit

The comments and scores of the previous Delphi round can be found below the indicators. Sometimes we adjusted the definitions based on these comments, sometimes we, after extensive discussion, decided not to incorporate the comments.

- Indicators with NEW in front of the definitions are newly proposed indicators based on the previous round
- All outcome indicators will be adjusted with a case-mix model (e.g. IMPACT or CRASH)

# Strongly disagree = 1 - strongly agree = 5

Please (re) rate the indicator(s)

## 1. NEW: Process: Median accident- to- ICU- admission time

Extra: Time of the accident/injury to ICU-door-time

Please choose the appropriate response for each item:

(5-point Likert scale):

|  | Strongly disagree | Disagree | Neither agree nor disagree | Agree | Strongly agree | I don’t know |
| --- | --- | --- | --- | --- | --- | --- |
| **Validity**: It is likely that better performance on the indicator reflects better processes of care and leads to better patient outcome | 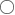 | 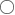 | 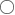 | 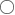 | 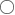 | 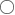 |
| **Discriminability**: It is expected that there is variability in clinical practice | 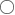 | 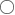 | 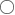 | 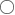 | 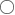 | 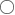 |
| **Feasibility**: Measurement of the indicator is feasible  (data for the indicator are available or easy to obtain) | 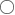 | 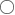 | 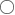 | 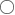 | 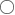 | 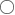 |
| **Actionability**: The indicator can be used to improve quality of care | 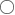 | 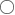 | 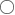 | 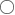 | 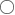 | 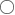 |

**Staff**

## 2. Structure: A daily meeting between intensivist and neurosurgeon to discuss patients with TBI at the ICU (yes/no)

Group scores previous Delphi round: median (min-max)

Validity = 4 (0-5), discriminability = 4 (1-5), feasibility = 4 (1-5), and actionability = 4 (1-5)

Comments previous Delphi round:

- *I think there should be a daily discussion between neurosurgeons and intensivists if patients are present. Or the neurosurgeon should be asked to join morning meetings or multidisciplinary meetings in the afternoon*
- *Neurosurgeon= neurologist*

## 3. Process: Number of visits by a neurosurgeon/ total number of ICU-days in patients with TBI

Group scores previous Delphi round: median (min-max)

Validity = 4 (2-5), discriminability = 4 (2-5), feasibility = 4 (1-5), and actionability = 4 (2-5)

Comments previous Delphi round:

- *This depends on the existing structure and cannot be answered as an indicator... in our ICU neurosurgeons will be asked for consultation if deemed necessary*

*- Neurosurgeon = neurologist*

**4. NEW: Structure: Total number of disciplines (i.e. neurologist, physiotherapy, occupational therapy) involved during ICU stay**

**CT scan**

## 5. Structure: 24/7 availability of a CT scan and radiologist review (yes/no)

Group scores previous Delphi round: median (min-max)

Validity= 5 (4-5), discriminability = 5 (2-5), feasibility = 5 (3-5), and actionability = 5 (2-5)

Comments previous Delphi round:

- 24/7 availability of radiologist physical presence
- A (neuro)radiologist review available 24/7

# ICP-monitoring

## 6. Process: Number of *severe* (GCS 3-8) TBI patients with ICP monitoring/ number of

***severe* TBI patients at the ICU**

Group scores previous Delphi round: median (min - max)

Validity = 4 (1-5), discriminability = 5 (3-5), feasibility 4 (2-5), and actionability = 4 (1-5)

Comments previous Delphi round:

- I would call this a process indicator (like percentage of patients who received thrombolysis in stroke care). For this indicator you could consider a minimum time of ICP monitoring per patient?
- Specific indications when or when not to monitor ICP are necessary, pure ICP monitoring may have side effects as a result of over treatment

## 7. Structure: 24/7 availability of a certified person at your center that can insert an ICP monitor within 2 hours after admission at the ICU (yes/no)

Group scores previous Delphi round: median (min-max):

Validity = 5 (2-5), discriminability = 4 (2-5), feasibility = 5 (1-5), and actionability = 4 (2-5)

Comments previous Delphi round:

- The 24/7 certified person is difficult - they may be present (easy to measure from records), but not available in practice (less measurable)

## 8. Process: Is the ventricular catheter levelled at the foramen of Monro according to a protocol? (yes/no)

Group scores previous Delphi round: median (min-max)

Validity = 4 (1-5), discriminability = 4 (1-5), feasibility = 4 (1-5), and actionability = 4 (1-5)

Comments previous Delph round:

- This is more a process indicator
- This is problematic as we have seen in CENTER. It should be posed as leveled at foramen M and not zerood. Which is a proces to zero a pressure dome and as long a patient heart and head are not a few 100 meters from eachother it makes no difference where it is zerood, except that is is done towards air pressure. Leveled at the head or heart has no consequence if the head is not raised, as it will mean te same thing
- Do you mean ventricular catheter or do you mean the ABP sensor? I think the later makes more sense..

## 9. Outcome: Number of EVD infections in patients with TBI/ total number of patients with TBI at the ICU with an EVD inserted

Extra: Only for centers that use ventricular catheters

Group scores previous Delphi round: median (min-max)

Validity = 4 (3-5), discriminabilty = 4 (2-5), feasibility = 4 (1-5), and actionability = 4 (3-5)

Comments previous Delphi round:

- EVD infections is very important but lacks a diagnostic gold standard and so much variation may be due to differences in antiobiotic prescribing and local criteria for defining infection
- This indicator obviously needs case-mix adjustment. Discriminablity depends on the incidence of these infections

# Deep Venous Thrombosis (DVT)

## 10. Process: Number of patients with TBI that receive any DVT prophylaxis/ total number of patients with TBI at the ICU

Extra: Timing (application of prophylaxis in days from the injury) and type of DVT prophylaxis (mechanical and/or pharmaceutical) can be registered as well

Group scores previous Delphi round: median (min-max)

Validity = 4 (2-5), discriminability = 4 (2-5), feasibility = 4 (2-5), and actionability = 4 (2-5)

Comments previous Delphi round:

- Need to specify this is *any* prophylaxis
- The info below the indicator is confusing

## 11. Process: Number of patients with TBI that receive mechanical DVT prophylaxis (e.g. stockings) initiated within 6 hours/ total number of patients with TBI at the ICU

Extra: Exclude patients with leg fractures

Group score previous Delphi round: median (min-max)

Validity = 4 (1-5), discriminability = 4 (2-5), feasibility = 4 (1-5), and actionability = 4 (1-5)

Comments previous Delphi round:

May be subject to variation if patient population has extracranial trauma (may be imposible with bilateral leg fractures for example). This may introduce center-center differences based on case-mix

## 12. Process: Number of patients with TBI at the ICU that receive pharmaceutical prophylaxis with low molecular weight heparins / total number of TBI patients admitted to the ICU

***This QI is about the choice of prophylaxis (low molecular weight heparin), not about timing.***

Group scores previous Delphi round:

Validity = 4 (2-5), discriminability = 4 (2-5), feasibility = 4 (2-5), and actionability = 4 (2-5)

Comments previous Delphi round:

- The vast majority get LMWH at *some* point but I doubt that is very valid for TBI outcome: it's the 72 hour time point where there is some variability

## 13. Outcome: Number of TBI patients with confirmed DVT during the first 7 days of ICU admission / total number of TBI patients at the ICU (exclude patients with DVT during presentation)

Group scores previous Delphi round: median (min-max)

Validity = 4 (2-5), discriminability = 4 (2-5), feasibility = 4 (1-5), and actionability = 4 (1-5)

Comments previous Delphi round:

- Confirmed DVT will be difficult to assess- many units will not scan. Confirmed PE within 1st week of admission may be a better measure - but need to exclude patients who present with PE. May still be institutional variation in threshols for scan however
- Routine ultrasound is possibly not done by anyone, anywhere
- I don't know of any units that routinely ultrasound legs

## 14. NEW: Structure: The timing of pharmacautical prophylaxis is reviewed daily and denoted in the medical record (yes/no)

Comments previous Delphi round:

- LMWH timing is also important. I suspect what is actually critical is that there is a process (and that it happens) to ensure this is reviewed regularly.

# Respiration and ventilation

## 15. Process: Number of TBI patients with the presence of abnormally low PaCO2 (<4 kPa) in at least one blood gas analysis during ICU stay/ number of patients with TBI at the ICU

Group scores previous Delphi round: median (min - max)

Validity = 4 (1-5), discriminability = 4 (2-5), feasibility = 4 (1-5), and actionability = 4 (2-5)

Comments previous Delphi round:

- Very hard to measure if not using an electronic system
- Should be better defined. Is it meant low CO2 (<4 kPa) at any time during ICU stay at least once?
- What is meant as prensence? At admission, during ICU stay?
- I'm not sure how to interprete this question. The answer is that I strongly disagree that patients should be hyperventilated
- ICP should be OK because of low PaCO2

# Glucose

# 16. Outcome: Number of TBI patients with any blood glucose below 4 mmol/L (hypoglycemia)/ number of TBI patients at the ICU

Group scores previous Delphi round: median (min-max)

Validity = 4 (3-5), discriminability = 4 (2-5), feasibility = 4 (1-5), and actionability = 4 (2-5)

Comments previous Delphi round:

- Episodes of hypoglycemia likely to be rare so although it varies, I think discrimination likely to be poor in reality
- May be difficult/ impossible to abtain accurate stats in all ICUs
- I don't think this is a specific indicator for TBI patients. Is more general for ICU care

# 17. NEW: Outcome: Number of TBI patients with any blood glucose above 10 mmol/ L (180 mg/ dL, hyperglycemia)/ total number of TBI patients at the ICU

**Nutrition**

**18. NEW: Process: Number of patients with start of (early) enteral nutrition within 72 hours/ number of patients with enteral feeding during ICU stay**

**Surgery**

## 19. Structure: The presence of a protocol or institutional guideline that provides indications for surgery with SDH and EDH (yes/no)

Extra: SDH: subdural hematoma, EDH: epidural hematoma Group scores previous Delphi round: median (min-max)

Validity = 4 (3-5), discriminability = 4 (2-5), feasibility = 4 (3-5), and actionability = 4 (2-5)

Comments previous Delphi round:

- It depends on what is in the protocol/ whether the protocol is evidence-based

## 20. Process: Number of decompressive craniectomies in TBI patients/ number of patients with TBI with increased ICP refractory to maximum osmotic agent treatment according to institutional guidelines

Group scores previous Delphi round: median (min-max)

Validity = 4 (2-5), discriminability = 4 (2-5), feasibility = 4 (2-5), and actionability = 4 (2-5)

Comments previous Delphi round:

- Difficult to define maximal osmotic Rx. It is unknown whether it represents optimal Rx (optimal timing undefined); it is not a good indicator in my opinion
- Agree craniectiomy can be useful, but this wording favours aggresive use
- Third tier therapies (e.g. brabiturates and DC) should be considered equivalent. The number of DCs in isolation would likely be a poor indicator: some may do too many, others too few
- Decompressive craniectomies worsen the outcome of survivors: I don't think it could be a useful indicator

## 21. NEW: Process: Median door-to-operation time for acute operation of SDH and EDH with surgical indication

Extra: SHD = subdural hematoma, EDH = epidural hematoma

**Paramedics**

## 22. Process: Number of TBI patients with a support plan (e.g. rehabiliation) after ICU discharge/ number of TBI patients discharged from the ICU

Extra: plan consists of physio-, speech-, and occupational therapist goals during hospital stay

Group scores previous Delphi round: median (min-max)

Validity = 5 (2-5), disccriminability = 5 (3-5), feasibility = 4 (2-5), and actionability = 4 (2-5)

Comments previous Delphi round:

- Suggest changing "rehabilitation plan" to "support plan (e.g. rehabilitation)"
- Do you need to refine this - I think it is important- but what standard of plan is needed?

**Assessment scales at the ICU**

## 23. Process: The number of assessments of consciousness (e.g. RASS or GCS) in patients with TBI at the ICU/ total number of ICU days in TBI patients

Extra: RASS= Richmond Agitation-Sedation Scale, GCS= Glasgow Coma Scale

Group scores previous Delphi round: median (min-max)

Validity = 4 (2-5), discriminability = 4 (3-5), feasibility = 4 (1-5), and actionability = 4 (1-5)

Comments previous Delphi round:

- Not possible to perform in sedated patients, only later in the ICU stay
- The nominator is number of days in which patients are assessable - i.e. deeply sedated. However, being able to categorize patients as assessable will be subjective..
- If a patient is sedated the GCS cannot be measured, but good care can be given with ICP measurement

## 24. NEW: Process: Number of assessments of pupillary responses/ total number of ICU days in patients with TBI

**25. NEW: Process: Number of assessments of motor scores of the GCS/ total number of ICU days in patients with TBI**

Extra: GCS = Glasgow Coma Scale

**In-hospital outcomes**

**26. Process: The median overall length of stay in the ICU of TBI patients**

Group scores previous Delphi round:

Validity = 4 (2-5), discriminability = 4 (2-5), feasibility = 4 (2-5), and actionability = 4 (2-5)

Comments previous Delphi round:

- Confounded by ICU bed pressure
- This really depends on case-mix, there might be large differences in TBI severity/ prognosis among patients with severe TBI at the ICU
- Length of stay could also be considered a process measure

## 27. Outcome: Number of in-ICU deaths among patients with TBI/ total number of ICU- admitted patients with TBI

Group scores previous Delphi round: median (min-max)

Validity = 4 (1-5), discriminability = 4 (2-5), feasibility = 4 (1-5), and actionability = 4 (1-5)

Comments previous Delphi round:

- This really depends on case-mix, there might be large differences in TBI severity/prognosis among patients with severe TBI at the ICU
- I would consider mortality at a certain time-point. How do you deal with patients transferred to another hospital?
- Hospital deaths could also derive from a decision of suspending futile therapies: it could hardly be considered an objective indicator
- TBI and ICU geared SMR needs weighting IMPACT variables and ISS, SAPS III or APACHE, and S100b 24-hour peak. In the ... Intensive Care registry we have seen that prognostic values of indicators change over time as providers adapt to them... even though processes and outcomes might not really have changed

## 28. Outcome: Incidence of ventilator associated pneumonia (VAP) in patients with TBI/ total number of TBI patients with mechanical ventilation at the ICU

Extra: Pneumonia defined as 'the presence of new lung infiltrate plus clinical evidence that the infiltrate is of an infectious origin, which includes the new onset of fever, purulent sputum, leukocytosis, and decline in oxygenation.'

VAP is defined as a pneumonia occuring >48 hours after endotracheal intubation

*American Thoracic Society; Infectious Diseases Society of America. Guidelines for the management of adults with hospital- acquired, ventilator-associated, and healthcare-associated pneumonia. Am J Respir Crit Care Med. 2005*

Group scores previous Delphi round:

Validity: 4 (1-5), discriminability = 4 (2-5), feasibility = 4 (2-5), and actionability = 4 (1-5)

Comments previous Delphi round:

VAP definition actually more difficult and has been problematic to operationalize in practice. US has changed to "ventilator associated events" instead. Will depend on unit case-mix (i.e. extracranial injuries in case-mix)

## 29. NEW: Outcome: Number of TBI patients with decubitus grade 2 or higher at the ICU/ number of TBI patients at the ICU

Extra (also register the grade):

Grade 1: Pressure zone with redness that does not blanch with fingertip pressure, with skin still intact

Grade 2: Decubitus ulcer (pressure sore) with skin erosion, blister, partial loss of the epidermis and/or dermis, or skin loss

Grade 3: Decubitus ulcer (pressure sore) with loss of all skin layers and damage or necrosis of the subcutaneous tissue, which may extend down to the underlying fascia

Grade 4: Decubitus ulcer (pressure sore) with necrosis of muscle, bone, or supportive structures such as tendons or joint capsules

# After discharge/follow-up outcomes

# 30. Process: Number of patients with TBI with a structured interview to assess functional outcome (e.g. Glasgow Outcome Scale - Extended) at follow-up (at least after 3 months)/ number of discharged patients with TBI and ICU stay who survived up to 3 months

Group scores previous Delphi round: median (min-max):

Validity = 4 (2-5), discriminability = 4 (3-5), feasibility = 4 (1-5), and actionability = 4 (2-5)

Comments previous Delphi round:

- "structural" = "structured". The item seems a bit specific to a particular assessment

## 31. Outcome: Total number of TBI patients that returned to work or school at 6 months/ number of TBI patients at work or school before injury

Group scores previous Delphi round: median (min-max)

Validity = 4 (2-5), discriminability = 4 (2-5), feasibility = 4 (1-5), and actionability = 4 (2-5)

Comments previous Delphi round:

- Extremely sensitive to case-mix and cause of ICU stay. Many patients with extracranial injuries after trauma may have minorTBI. Hard to adjust for this case-mix
- "Household activities" is much lower level than "work" and "school"

## 32. Outcome: Total number of TBI patients that returned to daily activities at 6 months/ number of TBI patients not at work or school before injury

Group scores previous Delphi round: median (min-max)

Validity = 4 (2-5), discriminability = 4 (2-5), feasibility = 4 (1-5), and actionability = 4 (2-5)

Comments previous Delphi round:

- Extremely sensitive to case-mix and cause of ICU stay. Many patients with extracranial injuries after trauma may have minorTBI. Hard to adjust for this case-mix
- "Household activities" is much lower level than "work" and "school"
- Suggest to replace "household activities" by "daily activities"

## 33. NEW: Outcome: Number of deaths after 6 months/ expected number of deaths based on a prediction model (e.g. IMPACT or CRASH)

**34. NEW: Process: Number of patients with neuropsychological testing at hospital discharge/ number of patients with TBI discharged from the hospital**

**35. NEW: Structure: Protocol available for family consult after a patient died at the intensive care unit (yes/no)**

**Outcome scales**

**The following indicators (GOSE, SF-36 and Qolibri) received the highest ranking in the previous Delpi round with a timing at 6 months, please rate the indicators to confirm validity, discriminability, feasibility, and actionability**

## 36. NEW: Outcome: The median score of the GOSE from all patients with TBI at 6 months/ number of patients with TBI discharged from the ICU

Extra: GOSE = Glasgow Coma Scale - Extended

**37. NEW: Outcome: The median score of the SF-36 from all patients with TBI at 6 months/ number of patients with TBI discharged from the ICU**

Extra: SF-36 = 36-item Short Form Survey

**38. NEW: Outcome: The median score of the QOLIBRI of all patients with TBI at 6 months/ number of patients with TBI discharged from the ICU**

Extra: QOLIBRI = Quality of Life after Brain Injury

## The following two indicators were proposed in the comments

**39**. **NEW: Outcome: The median score of the HADS or CES-D from all patients with TBI at 6 months/ number of patients with TBI discharged from the ICU**

Extra: HADS = Hospital Anxiety and Depression Scale, CES-D = Center for Epidemiological Studies Depression Scale (CES-D)

Comments from previous Delphi round:

- In our studies of longterm outcomes, the FAM (cognitive part of the FIM) and mood, which can be measured with for example the HADS or CES-D, are important predictors for employment outcome and health related quality of life

## 40. NEW: Outcome: The median score of the CSR-R from all patients with TBI who remain unresponsive at 6 months/ number of patient with TBI discharged or still in the hospital or in rehab

Extra: CSR-R = Coma Recovery Scale - Revised

Comments from previous Delphi round:

The outcome instrument list was difficult to complete as it took no account of level of recovery. So, for the patient who remains unresponsive, the CSR-R is the most important. Similarly different tests have different optimal timings - the CSR-R may be employed early in rehabilitation as patients emerge from coma, while the GOSE needs to be done as late as logistically feasible

# Final questions

## What do you consider as facilitators for implementation (multiple options):

Please choose **all** that apply:

###
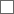
 Electronic data extraction (of quality indicators)


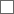
 External work force to collect data (so no administrative burden for your center)
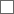
 One organization to oversee data collection (to increase uniformity)


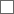
 Feedback on your performance


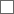
 Participation in trauma quality improvement programs
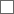
 Mandatory data collection


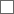
 Other:

**What do you consider as obstacles for implementation (multiple options):**

Please choose **all** that apply:
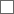
 Administrative burden
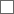
 Fear for publicity

###
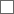
 Fear for future pay for performance
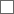
 Fear for benchmarking


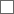
 Accountability (e.g. health insurance)
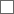
 Other:

**Do you have any comments on the questionnaire? Please specify when comments are related to specific quality indicators:**

Submit your survey.

Thank you for completing this survey.
