# Supplementary material for: Development of a quality indicator set to measure and improve quality of ICU care for patients with traumatic brain injury
Source: Crit Care. 2019 Mar 22;23:95. doi: 10.1186/s13054-019-2377-x (PMC6431034; doi:10.1186/s13054-019-2377-x)
Supplement: Supplementary file 4 — Indicator selection and scores during the Delphi process. (DOCX 66 kb) [file 13054_2019_2377_MOESM4_ESM.docx]

**Supplement 5. Indicator selection and scores during the Delphi process**

**Results Delphi round 1 (the advisory committee)**

| Red: removed from Delphi study  Orange: next Delphi round  * definition change in next round  (Validity, discriminability, feasibility, actionability) |
| --- |
| Protocol |
| 1. Structure: The existence of a protocol including specific guidelines (like the BTF guidelines) for Traumatic Brain Injury patients (yes/no) *  Median: 4; 4.5; 4; 4  IQR: 1; 1; 1; 2  2. Structure: The presence of (some form of) regular audits to check guideline adherence in your Intensive Care Unit (ICU) (yes/no)*  Median: 4; 4.5 4; 4  IQR: 0; 1; 0; 0  3. Structure: The presence of dedicated persons(s) to oversee guidelines development and maintenance at the ICU (yes/no)*  Mean: 4; 4; 4; 4  IQR: 1; 1; 1; 0 |
| Comments  - The BTF guidelines include only negative statements and no real indicators.  - I would very much doubt if any centre will have a dedicated person to oversee guidelines for TBI - perhaps in general, but not specific for TBI Suggest to re-phrase: The presence of dedicated persons(s) to oversee guidelines development and maintenance, including those for patients with TBI, at the ICU (yes/no)  - It would be important to be clear about whether the audit is specific to TBI (uncommon) or more general about processes of care, such as infection control policies, ventilator bundles etc (common). The latter may still be important - even though they are not specific for TBI care |
| Intensive Care Unit |
| 4. Structure: Does your hospital have a dedicated/specialized neurocritical care unit? (yes/no)  Median: 4; 4; 5; 4  IQR: 1; 1; 2; 2  5. Structure: Number of patients with ICP monitoring/ number of patients with indication for ICP monitoring *  *Based on the BTF guidelines*  indication for ICP monitoring: all salveagable patients with TBI and GCS 3-8 after resuscitation and an abnormal CT scan OR severe TBI patients with a normal CT AND 2 or more of 1) age >40 years, 2) unilateral or bilateral motor posturing (=M2 or M3), 3) SBP> 90 mmHg  Median: 4; 4; 4; 4  IQR: 1; 1; 2; 1  6. Structure: The availability of operating rooms 24 hours per day (yes/no) (after internal discussion it was decided to propose this indicator to the expert panel)  Median: 4; 3.5; 4; 4  IQR: 1; 1; 1; 1  7. Structure: Number of patients with TBI treated in your ICU annually  Median: 4; 5; 4; 3  IQR: 2; 1; 2; 2  8. Structure: Annual number of severe TBI patients GCS<8 on admission admitted to the ICU (volume)  Median: 4; 4; 4; 3  IQR: 2; 1; 2; 2  9. Structure: The presence of a step down unit where patients can still be monitored 24/7, but less intensively than at the ICU (yes/no)  Median: 4; 4; 4; 4  IQR: 1; 1; 2; 2 |
| Comments  Re Q 5: Were the indications for ICP monitoring not revised in the most recent edition?  For Q4 - The RAIN study showed that there was no difference in outcome between General ICUs in Neuro Centres and dedicated neuro ICUs - the label is not needed to ensure expertise  For Q5. The usage of ICP monitoring may be important in its own right, but is probably more likely a marker of quality of care - but increasing ICP monitoring in isolation may not improve care, unless the other factors that accompany its use in good quality centres are also implemented.  For Q6. OR availability 24 hours per day is important, and if not present, implementation could certainly improve care. However, I don't think this will have high discrimination - the vast majority of centres will have this  For Q7 and 8 , the issue is that though case volume is an important driver of care quality - it is not easily modifiable, at least at the level of individual hospitals. A change would require system level alterations Q9: Ticks all the boxes! |
| Staff |
| 10. Structure: Certified intensivist present in person 7 days a week during at least day-time (yes/no)  Median: 4.5; 4.5; 4.5; 4  IQR: 1; 1; 1; 1  11. Structure: Availability of a neurosurgeon (staff) 24/7 within 30 minutes after call (yes/no)  Median: 4.5; 4.5; 4.5; 4.0  IQR: 1; 1; 1; 1  12. Structure: Intensivist to ICU bed ratio  Median: 4; 4; 4; 4  IQR: 1; 1; 1; 1  13. Structure: ICU nurse to bed ratio  Median: 4; 4; 4; 4  IQR: 1; 1; 1; 1 |
| Comments  All good indicators, but for Q12 and Q13 there are no gold standard threshold figures, which when not met, have been shown to be associated with worse outcome |
| CT scan |
| 14. Structure: 24/7 availability of a CT scan (yes/no)  Median: 4.5; 4; 4.5; 4  IQR: 1; 2; 1; 1  15. Process: Performance of routinely repetitive CT scanning/ number of patients with severe TBI considered at high risk for deterioration (abnormal CT and/ or ICP monitoring at ICU)  Median: 4; 4; 4; 3  IQR: 2; 1 ;2;2 |
| Comments  For Q15 the difficulty will be defining the denominator - i.e. the number who would have been considered at high risk of deterioration - this is quite a subjective criterion |
| ICP-monitoring |
| 16. Structure: 24/7 availability of a certified person at your center that can insert an ICP monitor within 2 hours after admission at the ICU (yes/no)  Median: 4; 4; 4; 4  IQR: 1; 1; 1; 1  17. Structure: Is the ICP monitor zerood at the foramen of Monro in your clinic according to a protocol? Yes/no *  Median: 4; 4; 4; 4  IQR: 2; 1; 2; 2  18. Outcome: Number of EVD infections/ total number of patients with TBI at the ICU with an EVD inserted (after internal discussion with the coauthors it was decided to propose to the expert panel)  Median: 4; 4; 3; 4  IQR: 1; 1; 2; 3 |
| Comments  17 applies only to EVD Q 17: Incorrect grammar and typo: Is the ICP monitor is zerood  Q 18: Incorrect phrasing of Q: there will be many more patients with an EVD (and consequently more infections )for other indications than TBI. Hence, the equation is incorrect. Furthermore, only valid for centres than routinely monitor ICP by ventricular catheter.  For Q18, the difficulty will be defining EVD infections - will this be based on leukocyte count in CSF (sensitive but nonspecific) or positive cultures (specific but not sensitive) |
| Precautions ICP-monitoring |
| 19. Process: Antibiotics are given prior to ICP monitor insertion/ number of patients with TBI at ICU with ICP monitor  Median: 2.5; 4; 4.0; 2.5;  IQR: 2; 0; 1; 2  20. Process: Number of times a coagulation panel is assessed prior to insertion of an ICP monitor/ number of patients with TBI at the ICU and ICP monitor (after internal discussion with the coauthors it was decided to remove this indicator)  Median: 4; 4; 4; 4.5  IQR: 2; 1; 2; 2 |
| Comments  might be good to define "coagulation panel" in Q 20 Would this be a full coag panel, or only platelets and INR?  For Q19, I do not think that there are any good data that justify prophylactic antibiotics before placement of a parenchymal ICP probe, and infections are really rare with these |
| Sedatives |
| 21. Process: Number of patients with high dose barbiturate administration/ patients with refractory ICP in spite of maximum standard institutional medical (osmotic) and surgical (space occupying lesions extracted on admission) treatment  Median: 2.0; 4.0; 2.5; 1.5  IQR: 1; 1; 2; 2 |
| Comments  - Dubious question: high dose barbiturates are considered 3rd tier Tx However, high dose propophol will have equivalent effects. There is uncertainty on relative value/indication for barbs vs decompressive craniectomy  - The use of barbiturate infusions will vary inversely with use of hypothermia, decompressive craniectomy and/or ketamine sedation. I do not think this is a suitable quality indicator |
| Osmotic therapies |
| 22. Process: Number of patients with TBI receiving mannitol at doses of 0.25 to 1 g/kg body weight/number of TBI patients receiving mannitol  Median: 2.5; 3.5; 3.5; 2.5  IQR: 1; 1; 1; 1  23. Process: Number of TBI patients with a positive fluid balance > 1 L or negative fluid balance >-0.5 L (at day 1, 2 or 3) / number of patients with TBI at ICU  Median: 2.5; 3.0; 2.5; 2.5  IQR: 2; 1; 1; 1 |
| Comments  22 is probably: / number of patients with TBI at ICU  @ 22: I would replace Mannitol by Hyperosmolar fluids (eg including hypertonic saline. Currently, the Q makes little sense!)@ 23: Is a positive fluid balance > 1 L good or bad???Does calculation include perspiratio insensibilis?A negative fluid balance might be considered bad, but if this is post-operative, it may simply reflect correction of fluid load during anesthesia.....  The thresholds and dose of mannitol use will be confounded by use of hypertonic saline. Fluid balance is heavily dependent on other injuries - and not easy to reliably measureI think it would be more useful to ask whether osmotic therapies are employed maximally (for e.g to plasma osmolality 320 mosm/l, or Na >>150 before resorting to 3rd tier therapies (barbiturates, decompressive craniectomy) |
| Seizures |
| 24. Process: Number of patients with TBI receiving antiepileptic medication to prevent early seizures/ total number of patients with TBI at the ICU  Median: 2.5;2.5; 3.5; 2.5  IQR: 2; 0; 1;1 |
| Comments  Do you mean prophylactic anti-epileptic medication? Many centres in Europe will not routinely give anti-seizure prophylaxis - in contrast to US, where it is routine....Guidelines only state that med may reduce the occurrence of early seizures, but do not provide a risk/benefit perspective. All meds have side effects.... Evidence very weak and debatable  No strong data suggesting that early prophylactic AEDs improve outcome, or even reduce the incidence of early seizures - the latter particularly in units which use a lot of propofol |
| Fever |
| 25. Structure: Is there 24/7 sufficient availability of cooling devices to perform targeted temperature management for each patient with TBI at the ICU? (yes/no)  Median: 2.5; 4; 3.5; 3  IQR: 3; 2; 3; 2 |
| Comments  What would cooling devices be used for? A bit aggressive for treating fever...The Eurotherm trial showed an adverse effect of hypothermia...Not good as Quality Indicator  Could be refined to ask how many devices per 10 patients (had 0veral 5) |
| DVT |
| 26. Process: Number of patients with TBI that receive mechanical DVT prophylaxis (e.g. stockings)/ total number of patients with TBI at the ICU *  Median: 4; 4; 4; 4  IQR: 2; 1; 1; 2  27. Process: Number of patients with TBI at the ICU that receive medical prophylaxis with low molecular weight heparins / total number of TBI patients admitted to the ICU with (any) blood on CT *  Median: 4; 4; 4; 4  IQR: 1; 1; 1; 1  28. Process: Number of patients with TBI that receive medical prophylaxis with (low molecular weight) heparins within the first *72 hours* after admission/ total number of TBI patients admitted to the ICU with (any) blood on CT  Median: 3; 4; 3.5; 3  IQR: 2; 2; 2; 2  29. Outcome: Number of TBI patients with confirmed deep venous thrombosis or pulmonary embolism /total number of TBI patients at the ICU *  Median: 4; 4; 3; 4  IQR: 1; 0; 3; 1  30. Outcome: Number of patients with cerebral hemorrhage (any blood) on early CT/ total number of TBI patients at the ICU  Median: 2; 2; 2; 2  IQR: 2; 2; 3; 2 |
| Comments  Re 26/27: It is important if DVT prophylaxis is given or not - the "how" is less relevant For Quality assessment, you may need to combine 26 and 27@ 29: not quite clear: "Confirmed DVT": In centres routinely doing ultrasound of the legs, rate will be high. In those restricting ultrasound to clinical suspicion, rate will be low !@ 30: "any cerebral haemorrhage" would include tSAH: very poor QI  For Q 26 it would be useful to specify "mechanical prophylaxis initiated within 6 hours or admission"  For Q 27, it would be useful to specify "LMWH prophylaxis initiated within 5 days in the absence of a contraindication "Q 28 I'm not sure whether the implication here is that using LMWH within 72 hours in patients with traumatic ICH is a bad thing - if so - I don't agree. Arguable, the best practice would be to undertake repeat CT and start LMWH prophylaxis if there is no evidence of haematoma expansion after 48 hours - although, again, the evidence for this is very limited  Q29 is heavily dependent on the rate at which investigations are undertaken for DVT and PE - and is hence not a useful indicator. Mortality from PE would be a good indicator, but may be missed as a cause of death. This is an important variable, but difficult to collect accurately  Q 30 is badly phrased - it does not capture the spirit of preventable haemorrhage progression, and in any case, we do not have control of all of the possible factors that cause this. One additional QI might be the number of patients with clearly abnormal haemotatic parameters (INR >1.5, platelets) |
| Coagulopathy |
| 31. Structure: At your ICU is viscoelastic testing available for TBI patients? yes/no (after internal discussion with the coauthors it was decided to propose this indicator to the expert panel)  Median: 4; 4.5; 4; 3.5  IQR: 1; 1; 2; 1  32. Outcome: Number of patients with TBI with platelets below 75 x 109 /L at least in one lab results/ total number of patients with TBI at ICU  Median: 2.5; 3.5; 3.0; 2.5  IQR: 2; 2; 2; 1 |
| Comments  @ 32: Low platelets will likely depend on nature and severity of injury, and may not directly reflect Quality of Care  For Q 32 - the number of patients with severe thrombocytopenia will depend heavily on casemix - and be more common in units where major/multiple trauma and massive transfusion are more common - I don't think that, as asked, the metric is actionable. As in an earlier box, I think it would be more useful to find out whether haemostatic deficits are rapidly corrected (at least within the first 3 days post injury) |
| Respiration and ventilation |
| 33. Process: Number of TBI patients with a tracheostomy within 2 weeks after admission to the ICU/ number of patients with a tracheostomy placed during ICU stay (after internal discussion with the coauthors it was decided to propose this indicator to the expert panel)  Median: 4; 4; 3.5; 4.0  IQR: 1; 0; 2; 1  34. Process: Number of mechanical ventilated patients with TBI administered analgetics/ number of mechanical ventilated patients with TBI at the ICU *  Median: 3.5; 4; 3.5; 3.5  IQR: 2; 1; 3; 2  35. Outcome: Number of patients (in the absence of cerebral herniation) with partial pressure of carbon dioxide in arterial blood (PaCO2) outside normal range at any time / TBI patient-days at the ICU *  Median: 3; 3; 4; 3  IQR: 3; 2; 2; 3 |
| Comments  Q34 I think you mean analgesics  Outcome: Survival and GOSE?  @ 33: equation is wrong: need to include TBI patients in the denominator@ 34: How do you define "analgesics"? Would Paracetamol qualify as analgesic????@ 35: equation is wrong: would need TBI also in the first part! How do you define "normal" range? Many patiemnts may get mild hyperventilation as part of Tx. I suggest to delete this QI  For Q33 - this is reasonable, but it makes little sense to site tracheostomies in the presence of intracranial hypertension - but 2 weeks provides some but not perfect discrimination here  For Q 34 - I assume you mean opioids - if so we should specify For Q 35 - nearly every patient with intracranial hypertension will have a PaCO2 below the normal range, and it may be bad practice to move to some 2nd tier therapies without employing mild hyperventilation. Better questions might be the presence of abnormally low PaCO2 in the absence of intracranial hypertension (but difficult to record) |
| Red blood cell policy |
| 36. Outcome: Number of TBI patients with too low Hb (compared to protocol target level) at any time during ICU stay / total number of patients with TBI at ICU  Median: 3.5; 4.0; 2.5; 3.5  IQR: 2; 1; 2; 3  37. Outcome: Number of TBI patients with an arterial base deficit > 2 mmol/L below or above upper normal range limit within 48 hours after admission/ total number of TBI patients at the ICU  Median: 2.5; 4.0; 3.0; 2.5  IQR: 2; 1; 2; 1 |
| Comments  @ 36: Again, equation is incorrect! You need TBI also in first part. Further,"compared to protocol target level" is very unclear: protocols will differ by centre, and hence answers will be different and not comparable!@37: I am not aware that arterial base deficit has been studied in TBI  For Q 36 - what do you mean by too low a Hb? The guidelines suggest transfusion thresholds of 70 g/l, so this would be the guideline based threshold, but there are good reasons for transfusion at higher thresholds in the presence of intracranial hypertension. This will be a difficult QI to define rationally For Q 37 - I don't think this is valid |
| Glucose |
| 38. Structure: Do you have a protocol for glucose management available for patients with TBI at your ICU? yes/no  Median: 4; 4.5; 4; 4  IQR: 1; 1; 1; 1  39. Outcome: Number of lab results in patients with TBI with a blood glucose above the target level in your protocol/ number of TBI patients at the ICU *  Median: 4; 4; 4; 4  IQR: 3; 1; 3; 2 |
| Comments  @ 39: The absolute number will likely depend on the number of assays performed: in some centres this may be very frequent, in others maybe a few times per day Moreover, protocols will differ. Finally, not only the number, but the extent of deviation may be relevant  For Q 39 - I think you need number of patients with blood glucose above target range on more than two occasions within first 48 hours I would also ask for the number of patients with any blood glucose below 4 mmol/l - hypoglycaemia is demonstrably dangerous for sick brains and should be avoided |
| Nutrition |
| 40. Process: Number of TBI patients with basal full caloric replacement within 5 to 7 days post-injury / number of TBI patients at the ICU  Median: 4; 4; 4; 4  IQR: 2; 0; 1; 1  41. Process: Number of TBI patients with transgastric jejenual feeding/ number of patients with TBI at ICU * (after internal discussion it was decided to propose this indicator to the expert panel)  Median: 2.5; 4; 3.5; 2.5  IQR: 1; 1; 1; 1  42. Process: Number of TBI patients with basal full caloric replacement within 72 hours/ number of TBI patients at the ICU  Median: 3; 4; 3.5; 3  IQR: 0; 1; 1; 1 |
| Comments  should you maybe add a question on parenteral nutrition?  For Q 41 - transgastric jejunal feeding will depend on failed gastric feeding - and its use may be a good thing (if used to ensure feeding) or a bad thing (if other routine measures such as prokinetics and reduced opioid dose have not already been employed For Q 42 I am not aware of the literature that shows that establishing feeding within 72 hours is a process or outcome metric |
| Surgery |
| 43. Structure: The presence of a protocol/ institutional guideline that provides indications for surgery with SDH and EDH (yes/no)  Median: 4; 4; 4; 4  IQR: 1; 1; 0; 1  44. Process: Number of decompressive craniectomies/ number of patients with TBI with ICP refractory to maximum treatment dose with osmotic agents according to institutional guidelines (proposed to expert panel after internal discussion)  *Refractory high ICP: high ICP refractory to conventional first-tier therapies (CSF removal, mannitol, sedation, paralysis, mild hyperventilation*  Median: 3; 4; 4; 3  IQR: 2; 1;2 ; 2  45. Process: Number of large frontotemporoparietal decompressive craniectomies (not less than 12 x 15 cm or 15 cm diameter) / number of patients with decompressive craniecomies  Median: 3; 3; 3.5; 3  IQR: 2; 1; 2; 1 |
| Comments  @ 44: I don't think the ratio of DC in pat with refractory ICP can really be considered a good QI. There is little - if any - evidence showing it to be superior to high dose barbs. However, it is an interesting parameter@ 45: The essence is a "large DC" - Litt recommend at least > 100cm2: That would equate to diameter of 15 cm, but is much smaller than 12x15! |
| Paramedics |
| 46. Structure: A structural weekly meeting between intensivist and neurosurgeon to discuss TBI patients at the ICU (yes/no) *  Median: 4; 4; 4; 4  IQR: 1; 0; 1; 2  47. Process: Number of patients with TBI with prevention of contractures during ICU stay (using passive movements)/ total number of patients with TBI at the ICU admitted at least 2 weeks * (after internal discussion it was decided to propose this indicator to the expert panel)  Median: 4; 4; 3; 3.5  IQR: 4; 4; 4; 4  48. Process: Number of patients with TBI visited by a physiotherapist during ICU stay/ total number of patients with TBI at the ICU admitted at least 7 days* (after internal discussion it was decided to propose this indicator to the expert panel)  Median: 4; 4; 3.5; 4  IQR: 2; 1; 2; 2  49. Process: Number of patients with a rehabilitation plan after ICU discharge/ number of patients discharged from ICU  Median: 4; 4; 4; 4  IQR: 3; 1; 1; 2 |
| Comments  @ 46: a weekly meeting would not be enough! As such poor QI. Would need to be at least 2-3 times/week@ 49: For many patients, it will be too soon to determine Rehab plan....so, not really good QI@ 47/48: There is overlap here....Why different duration of adm for both Q: 2 weeks in 47, 1 week in 48Suggest to harmoniza duration  For Q 46 - difficult to record - what do you mean by structured meeting? also, 1/week is too infrequent For Q47 the question needs to be better articulated - either record the number of contractures, or the interventions used to prevent them - the current question conflates the two metrics ad is nor easy to answer For Q 48 the bar is too low - an ICU patient should be seen at least daily by a physiotherapist For Q 49 - it could be argued that the initial rehabilitation plan needs to be made within 48-72 hours of admission - but the question is reasonable as articulated |
| Assessment scales at the ICU |
| 50. Structure: Additional brain targeted monitoring (next to ICP/CPP and CO2) like PBrO2, SjO2, metabolic brain monitoring, NIRS, TCD etc/ (yes/no, how much modalities)*PbrO_2_: partial pressure of brain tissue oxygenSjO_2_: oxygen saturation of jugular bulb NIRS: near- infrared spectroscopy TCD: transcranial Doppler*  Median: 4; 4.5; 4; 3.5  IQR: 1; 1; 2; 1  51. Process: Number of TBI patients with daily assessments of the Glasgow Coma Scale (GCS) / number of TBI patients at the ICU*  Median: 4; 4; 4; 4  IQR: 1; 0; 2; 2  52. Process: Number of assessments of delirium presence with validated screening tool/ total number of ICU days in TBI patients *  Median: 4; 4; 4; 4  IQR: 1; 1; 1  53. Process: Daily visit by a neurosurgeon/ TBI patientdays at ICU*  Median: 4; 4; 4; 4  IQR: 1; 1; 1; 1  54. Process: Information on prognosis discussed with family by one of the treating physicians (ICU physician or neurosurgical physician) at least once/ total number of TBI patients at ICU *  Median: 4; 4; 4; 4  IQR: 1; 1; 2; 1 |
| Comments  @ 51: This will depend on severity and sedation for ventilation; the ratio is therefore not appropriate Further, once per day would not be enough Why not more simply: Daily assessments of the GCS?@ 52: Again, ratio not really appropriate: Can only assess delirium in patients who have regained consciousness!@ 53: I doubt if ratio is appropriate here. Suggest to simply ask: Daily visit by neurosurgeon@ 54: Again, ratio not appropriate Suggest to remove denominator  For Q 50 - I think that use of these ancillary monitors are markers of interest/expertise in TBI ICU care - but I have no evidence that implementing them in isolation (without a change in other management) will improve care For Q 51 - A daily GCS measurement in an ICU is too low a bar - if the patient needs ICU care GCS should be measured more frequently I would suggest at least 6 hourly (though I have no data to support this suggestion!)For Q 52 -It would not be sensible to check for delirium in patients who are deeply sedated for ICP control - so this needs to specify patients not in that categoryFor Q 53 - Some neurosurgeons visit, but do not document the visit if there is no disagreement with the intensivists management plan |
| Short term outcomes |
| 55. Process: Number of patients with TBI with a structural interview Glasgow outcome scale (extended) at hospital discharge/ number of discharged patients  Median: 3; 4; 3; 3  IQR: 1; 1; 2; 1  56. Process: Number of patients with TBI with a structural interview Glasgow outcome scale (extended) at follow-up (at least after 3 months)/ number of discharged patients  Median: 4; 4; 4; 4  IQR: 1; 1; 1; 1  57. Process: Number of patients with TBI discharged to a rehabilitation center/ total number of patients with TBI admitted to the ICU  Median: 3.5; 4; 4; 3.5  IQR: 2; 0; 1; 2  58. Outcome: The mean overall length of stay in the hospital of TBI patients  Median: 4; 4; 4; 4  IQR: 2; 1; 2; 2  59. Outcome: The mean overall length of stay in the ICU of TBI patients  Median: 4; 4; 4; 4  IQR: 2; 1; 2; 2  60. Outcome: Number of in-hospital deaths among patients with TBI/ total number of admitted patients with TBI  Median: 4; 4; 4; 4  IQR: 2; 1; 1; 2  61. Outcome: Incidence of ventilator associate pneumonia (VAP) in patients with TBI/ total number of TBI patients with mechanical ventilation*  Median: 4; 4; 3; 4  IQR: 2, 1, 2, 2  62. Outcome: Number of patients with TBI with severe sepsis or septic shock/ total number of patients with TBI at the ICU  Median: 4; 4; 4; 4  IQR: 1; 0; 2; 2  *Sepsis should be defined as life-threatening organ dysfunction caused by a dysregulated host response to infection. For clinical operationalization, organ dysfunction can be represented by an increase in the Sequential [Sepsis-related] Organ Failure Assessment (SOFA) score of 2 points or more, which is associated with an in-hospital mortality greater than 10%. Septic shock should be defined as a subset of sepsis in which particularly profound circulatory, cellular, and metabolic abnormalities are associated with a greater risk of mortality than with sepsis alone. Patients with septic shock can be clinically identified by a vasopressor requirement to maintain a mean arterial pressure of 65 mm Hg or greater and serum lactate level greater than 2 mmol/L (>18 mg/dL) in the absence of hypovolemia.*  *Singer et al. The Third International Consensus Definitions for Sepsis and Septic Shock (Sepsis-3) JAMA  2016* |
| Comments  I don't think any of these are useful unless they are rates % rather than numbers - and there is also adjustment for prognosis using an IMPACT or other model  @ 55: GOS(E) is an inappropriate outcome measure on discharge@ 57: Not clear if the first half of the equation relates to TBI patients who stayed at the ICU and if the Q relates to discharge destination from ICU or from Hospital@ 58/59: would need to be adjusted for injury severity; A large number of "milds" in the ICU will "contaminate" results@ 60: Good indicator, but would need to be adjusted for case-mix. SMR might be used, but has not been validated specifically for TBI!  For Q 55 - no evidence suggesting at GOSE is valid at ICU/hospital discharge For Q 59 and 58 - only valid if combined with mortality data For Q 61 - an agreed definition of VAP exists, but is difficult to implement, and centres can "game" the statistics |
| Outcome indicators: Long term outcomes |
| 63. Process: Number of patients with TBI receiving follow-up by a specialist within 2 months after discharge/ total number of patients with TBI discharged (not in rehab clinic)  Median: 4; 4; 4; 4  IQR: 2; 1; 2; 2  64. Outcome: The rate of brain complications at.. months  Definitions:  - When should the outcome indictor (brain complications) be measured? 3 months (0), 6 months(1), 12 months (2), 24 months (3), 3/6/12 months (4), 6/12 months (5), at all time points(6)  Median: 4; 4; 2.5; 4  IQR: 2; 1; 2; 2  Time: 4;6;0;2;1;1  65. Outcome: The rate of systemic complications at.. months  - When should the outcome indictor (brain complications) be measured? 3 months (0), 6 months(1), 12 months (2), 24 months (3), 3/6/12 months (4), 6/12 months (5), at all time points(6)  Median: 4; 4; 2.5; 4  IQR: 2; 1; 1; 2  Time: 4; 2; 0; 0; 0; 1  66. Outcome: Total number of patients that returned to work/school at .. months*  - When should the outcome indictor (brain complications) be measured? 3 months (0), 6 months(1), 12 months (2), 24 months (3), 3/6/12 months (4), 6/12 months (5), at all time points (6)  Median: 4; 4 ; 4; 4  IQR: 1; 1  Time: 5; 2; 1; 2; 1; 2 |
| Comments  Again % not numbers and adjusted for casemix /prognosis would make many of these useful  @ 63: would be less relevant (or perhaps inappropriate) for patients still in a rehab centre (and under control of physician there)@ 64: Too vague! Brain complications would need to be defined! Do you mean infection (meningitis/absces/empyema), hydrocephalus or subdural effusions, or also brain edema and raised ICP?@ 65: Too vague! systemic complications need to be defined. If you mean pneumonia, sepsis etc - the timing should be discharge from ICU. If you mean, for example, heterotopic ossification, it would be later. DVT/PE may only become evident after discharge@ 66: Your setting yourself up for being accused of gender discrimination! What about the housewife (or houseman) taking care of children etc at home and resuming household activities? |

**Results Delphi round 2 (expert panel)**

| Green: inclusion final set  Orange: Good agreement, but no consensus/ changed definition */ comments -> next round  Red: exclusion final set  * changed definition in next round  (Validity, discriminability, feasibility, actionability) |
| --- |
| Protocol |
| 1. Structure: The existence of a protocol including specific guidelines (like the BTF guidelines or institutional guidelines) for Traumatic Brain Injury patients (yes/no)  Median: 5, 4, 4, 4  IQR: 1, 1, 1, 1  2. Structure: The presence of (some form of) regular audits to check guideline adherence in general at the Intensive Care Unit (ICU) (yes/no)  Median: 4, 4, 4, 4  IQR: 1, 1, 1, 1  3. Structure: The presence of dedicated person(s) to oversee guidelines development and maintenance, including those for patients with TBI, at the ICU (yes/no)  Median: 4, 4, 4, 4  IQR: 1, 1, 1, 1  Comments: Feasability is the big issue especially concerning the question of adherence to guidelines even if they exist. |
| Intensive Care Unit |
| 4. Structure: Does your hospital have a dedicated/specialized neurocritical care unit? (yes/no)  Median: 5, 4, 5, 4  IQR: 1, 1, 1, 1.75  5. Structure: The availability of operating rooms 24 hours per day (yes/no)  Median: 5, 5, 5, 5  IQR: 0, 1, 0, 1  6. Structure: The presence of a step down unit where patients can still be monitored 24/7, but less intensively than at the ICU (yes/no)  Median: 4, 4, 5, 4  IQR: 1, 1, 1, 1.75  Comments :  - Actionability depends on the result/ outcome of the indicator.  - My Clinical work is in a rehab center and we do not have such facilities. So I took our academic hopspital as reference. If these structures are not available than it will be difficult to change this. Actionability therefore is low. |
| Staff |
| 7. Structure: Certified intensivist present in person 7 days a week during at least day-time (yes/no)  Median: 5, 5, 5, 5  IQR: 0, 1, 1, 1  8. Availability of a neurosurgeon (staff) 24/7 within 30 minutes after call (yes/no)  Median: 5, 5, 5, 5  IQR: 1, 1, 1, 1  9. Structure: Intensivist to ICU bed ratio  Median: 4, 4, 5, 4  IQR: 1, 1, 1, 1  10. Structure: ICU nurse to ICU bed ratio  Median: 5, 5, 5, 5  IQR: 1, 1, 1, 1  11. Structure: A structural meeting 2-3 times a week between intensivist and neurosurgeon to discuss TBI patients at the ICU (yes/no) *  Median: 4, 4, 4, 4  IQR: 1, 1, 1, 1  12. Process: Number of daily visits by a neurosurgeon/ total number of ICU days in TBI patients*  Median: 4, 4, 4, 4  IQR: 2, 1, 1, 1  Comments:  - Q 12 depends on the existing structure and cannot be answered as indicator... In our NICU neurosurgeons will be asked for consulation if deemed necessary....  - "Validity: It is likely that better performance on the indicator reflects better processes of care and leads to better patient outcome". By adding the word "and" you have this question hard to answer all over the questionnaire.  - I think there should be a daily discussion between neurosurgeons and intensivist if patients are present. Or the neurosurgeons should be ask to join morning meeting or multidisciplinary meeting in afternoon.  - Neurosurgeon = neurologist |
| CT scan |
| 13. Structure: 24/7 availability of a CT scan (yes/no) *  Median: 5, 5, 5, 5  IQR: 0, 1, 0, 1  Comments:  - Time to diagnostics/CT scanner/location of the CT scanner within the hospital  - 24/7 availability of radiologist physical presence  - And also a (neuro)radiologist review available 24/7 |
| ICP-monitoring |
| 14. Structure: Number of severe TBI patients with ICP monitoring/ number of severe TBI patients at the ICU *  Median: 4, 5, 4, 4  IQR: 1, 1, 1, 1  15. Structure: 24/7 availability of a certified person at your center that can insert an ICP monitor within 2 hours after admission at the ICU (yes/no)  Median: 5, 4, 5, 4  IQR: 1, 1, 1, 1  16. Structure: Is the ventricular catheter zeroed at the foramen of Monro according to a protocol? * Yes/no  Median: 4, 4, 4, 4  IQR: 1, 1, 1, 2  17. Outcome: Number of EVD infections in patients with TBI/ total number of patients with TBI at the ICU with an EVD inserted  Median: 4, 4, 4, 4  IQR: 1, 1, 1, 1  Comments:  - the 24/7 certified person is difficult- they may be present (easy to measure from rotas), but not available in practice (less measurable)EVD infections is very important but lacks a diagnostic gold-standard and so much variation may be due to differences in antibiotic prescribing and local criteria for defining infection.  - specific indications when or when not to monitor ICP are necessary* fi in pure DAIicp monitoring may have side effects as a result of over treatment  - Number of severe TBI patients with ICP monitoring/ number of severe TBI patients at the ICU: I would call this a process indicator (like percentage of patients who received thrombolysis in stroke care). For this indicator you could consider a minimum time of ICP monitoring per patient? Number of EVD infections in patients with TBI/ total number of patients with TBI at the ICU with an EVD inserted: this indicator obviously needs case-mix adjustment. Discriminability depends on the incidence of these infections.  - 16 is more a process indicator  - Question 16 is problematic as we have seen in CENTER1It should be posed as leveled at foramen M and not zeroed * which is a process to zero a pressure dome and as long a patients heart and head are not more than a few 100 meters from each other it makes no difference where it i zerod except that it is done towards air pressure.The data in CENTER has likely been interpreted differently by site2 leveled at the head or heart has no consequence if the head is not raised, as it will mean the same thing  - Do you mean ventricular cathether or do you mean the ABP sensor? I think the later makes more sense.... |
| Osmotic therapies |
| 18. Process: Number of TBI patients with osmotic therapies employed maximally (plasma osmolality 320 mosm/l, or Na up to 160 mmol/L) before resorting to 3rd tier therapies (barbiturates, decompressive craniectomy) /number of TBI patients with 3rd tier therapies (barbiturates, decompressive craniectomy)  Median: 4, 4, 4, 3  IQR: 1, 1, 1, 1  Comments:  - This is difficult- we do not know the threshold for starting 3rd tier therapies. More important that tiers are used in succession.  - barbiturates and craniectomy > evidence is lacking that it improves outcome  - I doubt whether this one is easy to understand or apply, and what it's added value is. I still had to read it several times, which may hamper usability of any indicator (should be easy as possible)  - I don't think this is a good indicator. Too vague. I think it is just one aspect of the whole intracranial hypertension protocol. And why so much emphasis on the osmotic therapy. There is no real evidence based literature available to prove its use.... |
| Deep Venous Thrombosis (DVT) |
| 19. Process: Number of patients with TBI that receive DVT prophylaxis/ total number of patients with TBI at the ICU *  *Extra: Timing (days from the injury) and type of DVT prophylaxis (mechanical and/or pharmaceutical) can be registered*  Median: 4, 4, 4, 4  IQR: 1, 1, 1, 1  20. Process: Number of patients with TBI that receive mechanical DVT prophylaxis (e.g. stockings) initiated within 6 hours / total number of patients with TBI at the ICU  Median: 4, 4, 4, 4  IQR: 1, 1, 1, 1  21. Process: Number of patients with TBI at the ICU that receive pharmaceutical prophylaxis with low molecular weight heparins / total number of TBI patients admitted to the ICU  *This QI is about the choice of prophylaxis (low molecular weight heparin), not about timing.*  Median: 4, 4, 4, 4  IQR: 0, 1, 1, 0.5  22. Outcome: Number of TBI patients with confirmed DVT /total number of TBI patients at the ICU *  *This QI will be adjusted for case-mix and mortality data and is only applicable in centers that routinely perform ultrasound of the legs*  Median: 4, 4, 4, 4  IQR: 2, 1.5, 2, 2  *Comments:*  - Q19- need to specify that this is *any* prophylaxis. Q20 is better.Q20- may be subject to variation if patient population has extracranial trauma (may be impossible with bilateral leg fractures for example). This may introduce centre-centre difference based on casemix. LMWH- timing is also important. I suspect what is actually critical is that there is a process (and that it happens) to ensure that this is reviewed regularly. We have clinical data for our patients- the vast majority get LMWH at *some* point but I doubt that is very valid for TBI outcome: it's the 72hour time point where there is some variability.22. Confirmed DVT will be difficult to assess- many units will not scan. Confirmed PE within 1st week of admission may be a better measure- but need to exclude patients who present with PE. May still be institutional variation in threshold for scan however.  - 19: the info below the question is confusing: i do not understand22: the info below the question is unexpected, Further, routine ultrasound is possibly not done by anyone, anywhere...  - In general: for all the outcome indicators, the discriminability depends (partly) on the incidence of the outcome, the outcome indicators need sound case-mix adjustment for analysis on between-center differences, and all (the most?) the outcome indicators lack a specific time-point.  - Regarding Question 22 - I don't know of any units that routinely ultrasound legs regularly |
| Coagulopathy |
| 23. Structure: At your ICU is viscoelastic testing available for TBI patients? yes/no  Median: 4, 4, 4, 3  IQR: 1.5, 1, 2, 1  Comments:  - We don't know, unfortunately, if this helps for certain.  - Only important at early stages of care  - Not entirely enthusiastic because viscoelastic testing may not be ready for prime-time use in neuroICU as yet - test is mature, but protocols are yet to come to terms with what we do with viscoelastic test results  - I am not sure about this indicator. Is there real benefit of this method for subacute care of TBI patients? |
| Respiration and ventilation |
| 24. Process: Number of TBI patients with a tracheostomy within 2 weeks after admission to the ICU/ number of TBI patients at the ICU  Median: 3, 4, 4, 3  IQR: 2, 1, 1, 2  25. Process: Number of mechanical ventilated patients with TBI administered opioids/ number of mechanical ventilated patients with TBI at the ICU  Median: 3, 4, 4, 3  IQR: 2, 1, 1, 2  26. Outcome: Number of patients with the presence of abnormally low PaCO2 (< 4kPa) in the absence of intracranial hypertension/ number of patients with TBI at the ICU *  *Adjusted for case-mix and mortality data*  Median: 4, 4, 4, 4  IQR: 2, 1, 1, 2  Comments:  - I'm not sure the relationship between tracheostomy and quality of care is simple. Different units will have very different thresholds.25. opioids difficult- multiple time points so hard to define / measure.26. Very hard to measure if not using an electronic system.  - 26: should be better defined. Is it meant low CO2,4kPa at any time during ICU stay at least once?  - Q 25 not easy to understand. Consider rephrasing Q26: what is meant as presence? At admission, during the ICU stay??  - Re Q 24: difficult to interoret as the ratio will depend on severity of patients. In practice, really only relevant to thos who have a severley disturbed level of consciousness at 2 weeks  - For Question 24 - the denominator is wrong - it should be one of the following (preferably the first):- the number of patients who eventually have tracheostomy over their ICU stay- The number of patients still intubated at 2 weeks post admission  - ICP could be OK because of low PaCO2  - Q 26. I'm not sure how to interpretate this question. The answer is that I strongly disagree that patients should be hyperventilated. |
| Glucose |
| 27. Structure: Do you have a protocol for glucose management available for patients with TBI at your ICU? yes/no  Median: 4, 4, 5, 4  IQR: 1, 1, 1, 1  28. Outcome: Number of TBI patients with any blood glucose below 4 mmol/L (hypoglycemia)/ number of TBI patients at the ICU  Median: 4, 4, 4, 4  IQR: 1, 1, 1.5, 1  Comments:  - Episodes of hypoglycaemia likely to be rare so although it varies, I think the discrimination likely to be poor in reality.  - re Q 28: may be difficult/impossible to obtain accurate stats in all ICUs  - Maintaining appropriate blood glucose levels (not above 180 mg/dl)Early enteral nutrition  - I don't think this specific indicator for TBI patients. Is more general for ICU care. |
| Nutrition |
| 29. Process: Number of TBI patients with basal full caloric replacement within 5 to 7 days post-injury / number of TBI patients at the ICU  Median: 4, 4, 4, 4  IQR: 1, 1, 0, 1  30. Process: Number of TBI patients with transgastric jejenual feeding to reduce ventilator associated pneumonia within 7 days after ICU admission/ number of patients with TBI at ICU  Median: 3, 4, 4, 3  IQR: 1, 1, 1, 1  Comments:  - Evidence base for Jej feeding poor.  - 30. Should the denominator be the number of ventilated TBI patients?  - Q 30 will depend on severity of case-mix: only relevant to patients who cannot feed themselves....  -Not convinced that the data for jejunal feeding satisfy using it as suggested  - This is more general ICU care. Not specific for TBI. |
| Surgery |
| 31. Structure: The presence of a protocol/ institutional guideline that provides indications for surgery with SDH and EDH (yes/no)  Median: 4, 5, 4, 4  IQR: 1, 1, 1, 2  32. Process: Number of decompressive craniectomies/ number of patients with TBI with increased ICP refractory to maximum osmotic agent treatment according to institutional guidelines  *Refractory high ICP: high ICP refractory to conventional first-tier therapies (CSF removal, mannitol, sedation, paralysis, mild hyperventilation*  Median: 4, 4, 4, 4  IQR: 1, 1, 1, 1  Comments:  - Difficult to define maximal osmotic Rx. It is unknown whether this represents optimal Rx (optimal timing undefined) so 32 is not a good indicator in my opinion.  - specific techniques used for craniectomy timing of sd,edh operationdoor to operation time for acute operation on sdh and edh  - 31 --> it depends on what is in the protocol / whether the protocol is evidence-based  - 32. Agree craniectomy can be useful, but this wording may favour aggressive use.  - Re Q 32: third tier therapies (eg barbiturates and DC) should be considered equivalent.The number of DCs in isolation would likely be a poor quality indicator: some centres may do too many, others too few  - Decompressive craniectomies worsen the outcome of survivors: I don't think it could be a useful indicator |
| Paramedics |
| 33. Outcome: Number of major contractures (hips, knee, ankle, shoulder, elbow, wrist) in TBI patients/ total number of patients with TBI at the ICU    This QI will be adjusted for case-mix and mortality data. *Contracture is defined as maximal passive range of motion of less than neutral position (e.g. for the ankle with knee in extension)*  Median: 4, 4, 3, 4  IQR: 2, 0.75, 2, 1  34. Process: Number of patients with TBI visited daily by a physiotherapist during ICU stay/ total number of patients with TBI at the ICU  Median: 4.5, 4, 4, 4  IQR: 1, 1, 1, 1  35. Process: Number of patients with a rehabilitation plan after ICU discharge/ number of patients  discharged from ICU *  Median: 5, 5, 4, 4  IQR: 1, 1, 1, 1  Comments:  - 33. this is interesting but I suspect it will be hard to capture from notes.35. Do you need to refine this- I think it is important but what standard of plan is needed?  - 33: info below question is not entirely clear to me  - 33. Outcome: Number of major contractures (hips, knee, ankle, shoulder, elbow, wrist) in TBI patients/ total number of patients with TBI at the ICU --> isn't this a case-mix variable?  - 35. suggest changing "rehabilitation plan" to "support plan (e.g. rehabilitation)"  - Re Q 33: only relevant to the more severely injured. Difficult/impossible to relate to total number without adjustment for case-mix |
| Assessment scales at the ICU |
| 36. Process: Number of daily assessments (3x or more) of the Glasgow Coma Scale (GCS)/ total number of ICU days in TBI patients *  Median: 4, 4, 4, 4  IQR: 1.5, 1, 1, 1  37. Process: Number of assessments of delirium presence with validated screening tool conscious TBI patients / total number of ICU days in conscious TBI patients  Median: 4, 4, 4, 4  IQR: 1, 1, 0, 0  38. Structure: Information on prognosis discussed with family by one of the treating physicians (ICU physician or neurosurgical physician) at least once during ICU stay  Median: 4, 4, 4, 4  IQR: 1, 1, 2, 2  Comments:  - 38. At what level of seniority? May be difficult to capture robustly if documentation poor.  - GCS// neurological examination is important where are the pupillary reactions, motor scores, lateralization  - 38 --> it reflects better processes of care but I don't think that it is directly associated with outcome  - 36 and 37, not possible to perform in sedated patients, only later in the ICU stay.  - For Question 36, the denominator is number of days in which patients were assessable - i.e. not deeply sedated. However, being able to categorise patients as assessable will be subjective....  - If a patient is sedated the gas cannot be measured, but good care can be given with icp measurement |
| Short term outcomes (in text beware: all these outcome indicators must be adjusted for casemix) |
| 39. Outcome: The median overall length of stay in the hospital of TBI patients  Median: 3, 4, 4, 4  IQR: 1, 1, 1, 1  40. Outcome: The median overall length of stay in the ICU of TBI patients  Median: 4, 4, 4, 4  IQR: 1, 1, 1, 1  41. Outcome: Number of in-hospital deaths among patients with TBI/ total number of admitted patients with TBI *  Median: 4, 4, 4, 4  IQR: 2, 1, 1, 2  42. Outcome: Incidence of ventilator associated pneumonia (VAP) in patients with TBI/ total number of TBI patients with mechanical ventilation at the ICU  *This QI will be adjusted for case-mix and mortality rates*  *Pneumonia was defined as the presence of “new lung infiltrate plus clinical evidence that the infiltrate is of an infectious origin, which includes the new onset of fever, purulent sputum, leukocytosis, and decline in oxygenation.”*  *VAP is defined as a pneumonia occurring >48 hours after endotracheal intubation.*  *American Thoracic Society; Infectious Diseases Society of America. Guidelines for the management of adults with hospital-acquired, ventilator-associated, and healthcare-associated pneumonia. Am J Respir Crit Care Med. 2005*  Median: 4, 4, 4, 4  IQR: 1, 0.25, 1, 1.5  43. Outcome: Number of patients with TBI with severe sepsis or septic shock/ total number of patients with TBI at the ICU  Median: 4, 4, 4, 4  IQR: 0.25, 1, 1, 2  Comments:  - 39. Will depend on what facilities are offered in the hospital (e.g. is there a fast access rehab facility in which case LOS will be expectedly longer).40. Confounded by ICU bed pressure.42. VAP definition actually more difficult and has been problematic to operationalize in practice. US has changed to 'ventilator associated events' instead. Will depend on unit case-mix (i.e. extracranial injuries in case-mix).  - 39,40,41: these really depend on case-mix, there might be large differences in TBI severity/prognosis among patients with severe TBI at the ICU  - Length of stay could also be considered as process measure.41. Outcome: Number of in-hospital deaths among patients with TBI/ total number of admitted patients with TBI--> I would consider mortality at a certain time-point. How do you deal with patients transferred to another hospital?  - Age and severity of injury adjustment  - Q 39-41 will depend on case mix. Results difficult to interpret in the absence of adjustment  - question 41Death % divided by risk adjusted population may be useful / a TBI specific SMR. As question is now ....you will adjust mortality with mortality rates ... do not follow  - Hospital deaths could also derive from a decision of suspending futile therapies: it could hardly be considered an objective indicator |
| Outcome indicators: Long term outcomes |
| 44. Process: Number of patients with TBI with a structural interview to assess the Glasgow Outcome Scale (extended) at follow-up (at least after 3 months)/ number of discharged patients with TBI and ICU stay who survived up to 3 months *  Median: 4, 4, 4, 4  IQR: 1, 1, 1, 1  45. Process: Number of patients with TBI receiving follow-up by a specialist within 2 months after discharge/ total number of patients with TBI discharged (not in rehab clinic)  Median: 4, 4, 4, 4  IQR: 1, 1, 2, 1  46. Outcome: Total number of patients that returned to work/school/household activities at .. months/number of TBI patients alive at … months *  Median: 4, 4, 4, 4  IQR: 1, 1, 1, 2  Overall Comments:  - 46. needs adjustment for case-mix  - 3,6,12 is to short to define final outcome but is still useful  - 46: depends on case-mix. You might add: corrected for all variables in the IMPACT model  - 44. "structural" = "structured". The item seems a bit specific to a particular assessment - suggest "Number of patients with TBI with an interview to assess functional outcome (e.g. the Glasgow Outcome Scale - Extended) at follow-up (at least 3 months or after)"46. "Household activities" is much lower level than "Work" and "school". Suggest " Total number of TBI patients that have returned to work/school at .. months/ number of TBI patients at work/ school before injury.  - Long term outcome > 24 months  - In our studies on longterm outcome:(E.Grauwmeijer et al.) - The FAM (the cognitive part of the FIM) and mood, which can be measured for example the HADS or CES-D, are important predictors for employment outcome and Health Related Quality of life.- For Health related Quality of Life the SF-36 can be used or the Quality of Life after Brain Injury (QOLIBRI)test.  - 46. extremely sensitive to case mix and cause of ICU stay . many patients with extra cranial injuries after trauma may have minor TBI. hard to adjust for this case mix. Few severe TBI patients will have returned within 2 months.  - I have been a bit wary about feasibility for all outcome variables - these can be challenging in TBI patients. However, even the ability to obtain follow up on patients may be a QI...  - Neuropsychiological testing? Also a talk with family if the patient died on the unit after 6 weeks. |

**Results Delphi round 3 (expert panel)**

| Red: exclusion final set: no consensus/ sensitivity analysis/ bad scoring  Green: inclusion final set  (Validity, discriminability, feasibility, actionability) |
| --- |
| Intensive Care Unit   1. NEW: Process: Median accident-to-ICU-admission time   Help: time of accident/injury to ICU-door-time  Median: 4,4,4,4  IQR: 1,1,1,2   1. Structure: A daily meeting between intensivist and neurosurgeon to discuss patients with TBI at the ICU (yes/no)   Median: 5,4,4,4  IQR: 1,1,1,1     1. Process: Number of visits by a neurosurgeon/ total number of ICU days in patients with TBI   Median: 4,4,4,4  IQR: 0,1,2,1   1. NEW : Structure: Total number of disciplines (i.e neurologist, physiotherapy, occupational therapy) involved during ICU stay   Median:4,4,4,4  IQR:1,1,1,1 |
| CT scan   1. Structure: 24/7 availability of a CT scan and radiologist review (yes/no)   Median: 5,4,5,5  IQR: 0,1,0,1 |
| ICP monitoring   1. Process: Number of severe TBI patients with ICP monitoring / number of severe TBI patients at the ICU   Median: 4,4,4,4  IQR: 1,1,1,1   1. Structure: 24/7 availability of a certified person at your center that can insert an ICP monitor within 2 hours after admission at the ICU (yes/no)   Median: 5,4,4,4  IQR:1,1,1,1   1. Process: Is the ventricular catheter leveled at the foramen of Monro according to a protocol? (Yes/no) (sensitivity analysis)   Median: 4,4,4,4  IQR:1,1,1,2   1. Outcome: Number of EVD infections in patients with TBI/ total number of patients with TBI at the ICU with an EVD inserted   Extra: adjusted with a case mix model (e.g. IMPACT or CRASH)  Median: 4,4,4,4  IQR: 1,1,0,1 |
| DVT prophylaxis   1. Process: Number of patients with TBI that receive any DVT prophylaxis/ total number of patients with TBI at the ICU   Extra: Timing (application of prophylaxis in days from the injury) and type of DVT prophylaxis (mechanical and/or pharmaceutical) can be registered as well  Median: 4,4,4,4  IQR: 1,1,1,0   1. Process: Number of patients with TBI that receive mechanical DVT prophylaxis (e.g. stockings) initiated within 6 hours / total number of patients with TBI at the ICU with the possibility to receive stocking   Extra: exclude patients with leg factures  Median: 4,4,4,4  IQR: 0,0,2, 0   1. Process: Number of patients with TBI at the ICU that receive pharmaceutical prophylaxis with low molecular weight heparins / total number of TBI patients admitted to the ICU   This QI is about the choice of prophylaxis (low molecular weight heparin), not about timing.  Median: 4,4,4,4  IQR: 0,0,1,0   1. Outcome: Number of TBI patients with confirmed DVT during the first 7 days of ICU admission /total number of TBI patients at the ICU (exclude patients with DVT during presentation) (sensitivity analysis)   Median: 4,4,4,4  IQR: 1,0,1,1   1. NEW: Structure: The timing of pharmaceutical prophylaxis is reviewed daily and denoted in the medical record (yes/no) (sensitivity analysis)   Median: 4,4,4,4  IQR: 1,1,1,2 |
| Respiration and ventilation   1. Process: Number of patients with abnormally low PaCO2 (< 4kPa) in at least one blood gas analysis during ICU stay/ number of patients with TBI at the ICU   Median: 4,4,4,4  IQR: 2,1,2,2 |
| Glucose   1. Outcome: Number of TBI patients with any blood glucose below 4 mmol/L (hypoglycemia)/ number of TBI patients at the ICU   Median: 4,4,4,4  IQR:1,1,0,1   1. NEW: Outcome: Number of TBI patients with any blood glucose above 10 mmol/L (180mg/dL, hyperglycemia)/ total number of TBI patients at the ICU   Median: 4,4,4,4  IQR: 0,0,0,1 |
| Nutrition   1. NEW: Process: Number of patients with start of (early) enteral nutrition within 72 hours/ number of patients with enteral feeding during ICU stay   Median: 4,4,4,4  IQR: 0,0,0,1 |
| Surgery   1. Structure: The presence of a protocol/ institutional guideline that provides indications for surgery with SDH and EDH (yes/no)   Median: 4,4,4,4  IQR: 1,1,1,0   1. Process: Number of decompressive craniectomies/ number of patients with TBI with increased ICP refractory to maximum osmotic agent treatment according to institutional guidelines   Median: 4,4,4,3  IQR: 1,1,1,1   1. NEW: Process: Median door-to-operation time for acute operation of sdh and edh with surgical indication   Median: 4,4,4,4  IQR: 1,1,1,1 |
| Paramedics   1. Process: Number of patients with a support plan (e.g. rehabilitation) after ICU discharge/ number of patients discharged from ICU   *Extra*: Plan consists of physio -, speech -, occupational therapist goals during hospital stay  Median: 5,5,4,4  IQR:1,1,1,1  Assessment scales at the ICU   1. Process: The number of assessments of consciousness (e.g. RASS or GCS) in patients with TBI at the ICU / total number of ICU days in TBI patients   Median: 4,4,4,4  IQR: 2,1,1,1   1. NEW: Process: Number of assessments of pupillary responses/ total number of ICU days in patients with TBI   Median: 4,4,4,4  IQR: 1,1,0,1   1. NEW: Process: Number of assessments of motor scores of the GCS/ total number of ICU days in patients with TBI   Median: 4,4,4,4  IQR: 1,1,0,1 |
| In-hospital outcomes   1. Process: The median overall length of stay in the ICU of TBI patients   Median: 4,4,4,3  IQR: 1,1,1,1   1. Outcome: Number of in-ICU deaths among patients with TBI/ total number of ICU-admitted patients with TBI   Median: 4,4,4,4  IQR: 1,1,1,1     1. Outcome: Incidence of ventilator associated pneumonia (VAP) in patients with TBI/ total number of TBI patients with mechanical ventilation at the ICU   Median: 4,4,4,4  IQR: 1,0,1,0   1. NEW: Outcome: Number of TBI patients with decubitus grade 2 or higher at the ICU/ number of TBI patients at the ICU   Median: 4,4,4,4  IQR: 1, 0.25, 0, 1 |
| After discharge/ follow-up outcomes   1. Process: Number of patients with TBI with a structured interview to assess functional outcome (e.g. the Glasgow Outcome Scale -Extended) at follow-up (at least after 3 months)/ number of discharged patients with TBI and ICU stay who survived up to 3 months (sensitivity analysis)   Median: 4,4,4,4  IQR: 1,1,1,1   1. Outcome: Total number of patients that returned to work or school at 6 months/number of TBI patients at work/school before injury (sensitivity analysis)   Median: 4,4,4,4  IQR: 1,1,0,2   1. Outcome: Total number of patients that returned to daily activities at 6 months / number of TBI patients not at work/school before injury (sensitivity analysis)   Median: 4,4,4,4  IQR:1,1,0,2   1. NEW: Outcome: Number of deaths after 6 months / expected number of deaths based on a prediction model (e.g. CRASH or IMPACT) (sensitivity analysis)   Median: 4,4,4,4  IQR: 1,1,1,0   1. NEW: Process: Number of patients with neuropsychological testing at hospital discharge/ number of patients with TBI discharged from the hospital   Median: 4,4,4,4  IQR: 1,1,1,1   1. NEW: Structure: Protocol available for family consult after a patient died at the intensive care unit (yes/no) (sensitivity analysis)   Median: 4,4,4,4  IQR: 1,0,1,1 |
| Outcome scales  The following indicators received the highest ranking (for inclusion in the final indicator set) in previous questionnaire with a timing at 6 months, please rate the indicators to confirm   1. NEW: Outcome: The median score of the GOSE from all patients with TBI at 6 months / number of patients with TBI discharged from the ICU   Median: 4,4,4,4  IQR: 1,1,0,1   1. NEW: Outcome: The median score of the SF-36 from all patients with TBI at 6 months/ number of patients with TBI discharged from the ICU and alive at 6 months   Median: 4,4,4,4  IQR: 0,1,1,1   1. NEW: Outcome: The median score of the Qolibri from all patients with TBI at 6 months/ number of patients with TBI discharged from the ICU and alive at 6 months   Median: 4,4,4,4  IQR: 2,1,1,1   1. NEW: Outcome: The median score of the HADS or CES-D from all patients with TBI at 6 months/ number of patients with TBI discharged from the ICU and alive at 6 months   Median: 4,4,4,3  IQR: 1,1,1,1   1. NEW: Outcome: The median score of the CSR-R from all patients with TBI who remain unresponsive at 6 months/ number of patients with TBI discharged or still in the hospital or in rehab alive at 6 months   Median: 4,4,4,3  IQR: 2,1,1,1 |
| Overall comments (most of the indicators that received these comments were already removed based on scoring). However, in this final round the scoring of the group outweighed individual comments)  - Gaming has been seen in the VAP literature- the definition of VAP is ambiguous and so has been interpreted to improve outcomes. This could apply to a number of quality indicators for TBI also.  - Q 16-18 are confusing. This because one may interpret the alternatives as good (glucose)  - Q 35: neuropsychological testing will not be feasible in all patients at discharge - therefore poor indicatorQ 32: This Q has a discriminatory flavor to it: It would seem that patients not at work or school before play are seen as "second level" citizens! What about housewife? retired people??Delete!Q 24 & 26: overlap! Both assess the level of consciousnessQ 24, 25 and 26: I have doubts about this. The preferred number of assessments will depend on the time after injury and the condition of the patient. In the early phase more frequent assessments are indicated.In patients heavily sedated and ventilated, the clinical monitoring is primarily done with ICP. In contrast, in patients not requiring ICP monitoring, frequent assessments are indicated.The denominator is further confounded by extracranial injuries being the reason for staying in the ICU...Q 15: What is the sense of reviewing timing daily once prophylaxis has been implemented?  - An important restriction of this questionnaire is the mono-disciplinary focus. Traumatic Brain Injury can of course be isolated, but is often 'just' one of the injuries a patient sustains. Any (outcome) indicator that is used in polytraumatized patients will result in biased, non-causative relationships. E.g. the percentage of (ventilator-associated) pneumonias in TBI patients will increase if the number of rib fractures is high. Collaboration and/or integration with existing 'holistic models' used to assess quality of polytrauma care (including TBI) is more or less mandatory.Secondly, 'TBI' is a container of many different diagnoses. Per individual diagnosis per individual patient outcome can be strongly depending on anatomical location or size of the injury. Finally, if goals are not established prior to any measurement, a lot of indicators become invaluable. If you want to measure patient safety, you need other indicators than if you want to get informed about e.g. efficiency of care. I think that lack of established goals prior to implementation leading to erroneous (public) conclusions fears most health care professionals. |
